# PNAC: a protein nucleolar association classifier

**DOI:** 10.1186/1471-2164-12-74

**Published:** 2011-01-27

**Authors:** Michelle S Scott, François-Michel Boisvert, Angus I Lamond, Geoffrey J Barton

**Affiliations:** 1 Division of Biological Chemistry and Drug Discovery, College of Life Sciences, University of Dundee, Dow Street, Dundee DD1 5EH, UK; 2 Wellcome Trust Centre for Gene Regulation and Expression, College of Life Sciences, University of Dundee, Dow Street, Dundee DD1 5EH, UK

## Abstract

**Background:**

Although primarily known as the site of ribosome subunit production, the nucleolus is involved in numerous and diverse cellular processes. Recent large-scale proteomics projects have identified thousands of human proteins that associate with the nucleolus. However, in most cases, we know neither the fraction of each protein pool that is nucleolus-associated nor whether their association is permanent or conditional.

**Results:**

To describe the dynamic localisation of proteins in the nucleolus, we investigated the extent of nucleolar association of proteins by first collating an extensively curated literature-derived dataset. This dataset then served to train a probabilistic predictor which integrates gene and protein characteristics. Unlike most previous experimental and computational studies of the nucleolar proteome that produce large static lists of nucleolar proteins regardless of their extent of nucleolar association, our predictor models the fluidity of the nucleolus by considering different classes of nucleolar-associated proteins. The new method predicts all human proteins as either nucleolar-enriched, nucleolar-nucleoplasmic, nucleolar-cytoplasmic or non-nucleolar. Leave-one-out cross validation tests reveal sensitivity values for these four classes ranging from 0.72 to 0.90 and positive predictive values ranging from 0.63 to 0.94. The overall accuracy of the classifier was measured to be 0.85 on an independent literature-based test set and 0.74 using a large independent quantitative proteomics dataset. While the three nucleolar-association groups display vastly different Gene Ontology biological process signatures and evolutionary characteristics, they collectively represent the most well characterised nucleolar functions.

**Conclusions:**

Our proteome-wide classification of nucleolar association provides a novel representation of the dynamic content of the nucleolus. This model of nucleolar localisation thus increases the coverage while providing accurate and specific annotations of the nucleolar proteome. It will be instrumental in better understanding the central role of the nucleolus in the cell and its interaction with other subcellular compartments.

## Background

The nucleolus was initially characterised over four decades ago and shown to be the site of ribosome subunit production [1]. It is now known to play a role in other cellular activities, including assembly of diverse ribonucleoprotein particles (RNPs), cell cycle progression and proliferation regulation, as well as the response to numerous forms of cellular stress [2-6]. All of the proteins that are strongly enriched in the nucleolus, including marker proteins such as fibrillarin, can nonetheless cycle continually in and out of the nucleolus, as discovered by photobleaching experiments [7]. In addition, many of the processes that occur, at least in part, in the nucleolus require the re-location of proteins to this nuclear sub-compartment. Many proteins are able to conditionally relocate between either the nucleoplasm, or other nuclear sub-compartments and the nucleolus [3,4]. In addition to the 'part-time' nucleolar proteins which remain in the nucleus, many proteins are known to travel between the cytoplasm (including cytoplasmic organelles) and the nucleolus. These include ribosomal and non-ribosomal proteins that travel to the nucleolus for assembly into ribosome subunits and other RNPs respectively, as well as many growth factors and cell cycle regulators [2,4,8]. The nucleolus thus accommodates a large amount of traffic and its composition is very dynamic, which may be facilitated by its lack of a surrounding membrane [6].

Recent large-scale proteomics experiments have detected thousands of distinct proteins that stably co-purify with nucleoli isolated from human cells [9-11]. Although the first datasets defining the nucleolar proteome did not offer information regarding the proportion of each of these proteins in the nucleolus relative to other cellular compartments, this information has now been obtained in a high throughput manner using a combination of cellular fractionation and SILAC protocols [12]. These data indicate that although thousands of distinct proteins are detected in the nucleolus, their degree of association with the nucleolus is variable. Some proteins are predominantly nucleolar while others, although detected in small numbers in the nucleolus and annotated as such in large databases, are present in much larger numbers in other cellular compartments. These proteomics data give a snapshot of the content of the nucleoli of a population of one cell type under specific conditions. In comparison to the first nucleolar proteome datasets [9-11], they provide a much clearer picture of the dynamic protein content of the nucleolus and its relationship with other cellular compartments. This methodology also offers the possibility of distinguishing the nucleolar-enriched proteins from the proteins which cycle between the nucleolus and other cellular locations or conditionally localise to the nucleolus. However, because only one cell type and a small number of conditions have been examined so far and because of the current limitation of the methodology, which does not yet offer full proteome coverage, the dynamic nucleolar proteome still has not been fully defined. Here, we investigate how a computational method can help fill this gap.

The prediction of eukaryotic protein subcellular localisation has been extensively investigated over the past decade using various machine learning methods and based on many diverse protein characteristics (reviewed in [13]). However, while many such predictors exist, most do not consider the nucleolus as a separate localisation: very few whole-cell predictors include the nucleolus in the list of cellular compartments to which they predict localisation [14-17]. Several nuclear-centric mammalian protein localisation predictors have been created to predict membership to one of at least four nuclear sub-compartments including the nucleolus [18-22]. However, proteins annotated as being in more than one subnuclear compartment are often not considered, thus substantially decreasing their actual coverage of the nuclear proteome. Because the individual nuclear subcompartments are not membrane-enclosed, it is expected that a significant proportion of nuclear proteins diffuse between these subcompartments and will be detected and annotated as present in several of these compartments. Thus these nuclear-centric predictors likely do not realistically model localisation patterns of nuclear proteins.

The prediction of nucleolar protein localisation has been investigated mainly in the context of a binary classification problem where proteins are predicted to be either associated with the nucleolus, or not. Such studies include a predicted nucleolar complex dataset, which is based on the clustering of protein-protein interactions, involving human proteins either detected experimentally in the nucleolus, or predicted to be nucleolar using a neural network [23]. More recent studies include a naïve Bayesian classifier trained to predict yeast nucleolar proteins and ribosomal components [24], a sequence-based support-vector machine predictor that differentiates between nucleolar associated and non-nucleolar associated nuclear mammalian proteins [25] as well as a kernel canonical correlation analysis predictor based on genomic sequence and protein-protein interaction data that also differentiates between nucleolar associated and non-nucleolar associated nuclear mammalian proteins [26].

**Figure 1:**
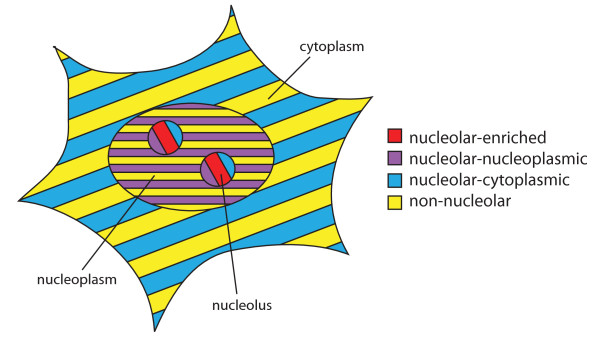
**Protein nucleolar association classes considered**. PNAC classifies human proteins into four distinct classes according to their degree of nucleolar association. The nucleolar-enriched protein group (red) consists of proteins that are predominantly nucleolar in all cell types and conditions. The nucleolar-nucleoplasmic group (purple) is composed of proteins identified in both the nucleolus and any other nuclear region. The nucleolar-cytoplasmic group (blue) consists of cytoplasmic proteins that also can localise to the nucleolus. The non-nucleolar group (yellow) comprises all proteins that never localise to the nucleolus. The non-nucleolar proteins can localise to other regions of the nucleus, the cytoplasm, plasma membrane or extracellularly.

Recent efforts to predict nucleolar association acknowledge the fluidity of the nucleolus and its close relationship with other cellular regions, but do not model different degrees of protein association with the nucleolus. In order to build on previous efforts, we investigate here the possibility of classifying the degree of nucleolar association of human proteins, by integrating various genomic and protein features in a Bayesian framework. More precisely, we predict whether proteins are highly nucleolar-enriched, highly non-nucleolar, nucleolar-nucleoplasmic or nucleolar-cytoplasmic (see Figure 1). The last two groups include proteins that localise to other cellular regions and cycle to the nucleolus or relocate to the nucleolus under specific conditions. To perform this classification, we consider several protein features including the frequency of specific amino acids in the protein sequence, the predicted presence of signal peptides, mitochondrial targeting peptides and nucleolar localisation sequences as well as expression data, Gene Ontology (GO) annotations and subcellular localisation annotations of protein interactors.

## Results and Discussion

### Architecture of the protein nucleolar association classifier

The Protein Nucleolar Association Classifier (PNAC) was created to investigate the classification of the degree of nucleolar association of human proteins into four classes as illustrated in Figure 1:

-**nucleolar-enriched **proteins which correspond to proteins that are accumulated predominantly in the nucleolus and likely include the core nucleolar proteins.

-**nucleolar-nucleoplasmic **proteins which can localise both to the nucleolus and to other nuclear regions. They either cycle between the nucleolus and other nuclear regions or are mainly nucleoplasmic but relocate to the nucleolus under specific conditions.

-**nucleolar-cytoplasmic **proteins which can localise to the nucleolus and the cytoplasm (or even extracellularly). They either localise to the nucleolus for assembly into larger complexes but then function mainly in the cytoplasm, cycle between these compartments, or are predominantly cytoplasmic but relocate to the nucleolus under specific conditions.

-**non-nucleolar **proteins which show little or no localisation in the nucleolus.

Because such detailed annotations describing dynamic characterisations of proteins are rarely available in large public databases, extensive manual curation of the nucleolus literature was required to create the datasets for this study. Additional File 1 shows experimentally determined proteins belonging to the three nucleolar-association classes (the nuclear-enriched, nucleolar-nucleoplasmic and nucleolar-cytoplasmic classes).

PNAC integrates diverse types of protein features and annotations in a naïve Bayes framework to classify human proteins according to their degree of nucleolar association. The individual features considered are summarised in Table 1 and detailed in the Methods section. As indicated in Table 1, five distinct features are taken into account: amino acid frequency, protein targeting motifs, gene co-expression, GO biological process and molecular function annotations as well as subcellular localisation annotations of interactors. Protein-protein interaction data have been included because many proteins are likely to be either targeted to, or retained in, specific cellular compartments by binding to proteins resident in these compartments. Since the nucleolus is not enclosed by a membrane, interaction based localisation could be widely employed by proteins associated with this compartment [6].

**Table 1:** Features considered in the prediction of nucleolar association

Features	Data source	Description	Bins
Amino acid frequency	Protein sequences from IPI [27]	PNAC considers the relative proportion of leucine, isoleucine, lysine and serine residues	5 bins for each distinct amino acid considered

Targeting motifs	Phobius [32], TargetP[33], NoD [34]	The predicted presence of signal peptides, transmembrane domains (TMDs), mitochondrial targeting peptides and nucleolar localisation sequences (NoLSs)	9 bins detailed in the Methods

Gene co-expression	GDS596 from the Gene Expression Omnibus [42]	The average Pearson correlation of expression between the query protein and proteins in the nucleolar-cytoplasmic training group using expression profiles from 79 physiologically normal tissues [35]	5 bins

GO	EBI Gene Ontology (GO) annotations [36] for human	Biological process and molecular function Gene Ontology (GO) annotations for the query protein are compared to those of the training set proteins	4 bins

Subcellular localisation of interactors	HPRD [31], Uniprot [30], IntAct [39] and PIPs [37,38] subcellular localisation annotations and/or protein interactions	A nucleolar proximity score is calculated for all the interactors of the query protein	5 bins

Each predictive feature is used to calculate a score for the presence of a given protein in each of the nucleolar association classes described above. For each feature, the association score for a given class is evaluated by considering the relative proportion of proteins from that class that have a specific state (i.e. that fall in a particular bin of that feature). The features are trained on manually curated nucleolar datasets (listed in Additional File 1) and a randomly generated non-nucleolar dataset. The final classification results from the product of the initial class priors and the scores derived from the individual predictive features as detailed in the Methods section. Proteins are annotated as belonging to their highest scoring class.

### Statistical tests of accuracy

#### Cross validation analysis

The classifier was first tested using a leave-one-out cross-validation test as described in the Methods. Table 2 (Test set 1 columns) shows the sensitivity and positive predictive values (PPV) for all classes. All classes obtain values significantly higher than those that would be obtained by random assignment (which are shown in the 'prior' column in Table 2). The overall accuracy of the predictor by leave-one-out cross validation is 0.86.

**Table 2:** Tests of accuracy

Class	Priors	Counts			**Sensitivity**^**d**^			**PPV**^**e**^		
		
		**Test set 1**^**a**^	**Test set 2**^**b**^	**Test set 3**^**c**^	Test set 1	Test set 2	Test set 3	Test set 1	Test set 2	Test set 3
Nucleolar-enriched	0.20	30	15	52	0.72	0.78	0.56	0.68	0.61	0.47

Nucleolar-nucleoplasmic	0.15	22	7	24	0.73	0.57	0.50	0.63	0.60	0.41

Nucleolar-cytoplasmic	0.15	24	16	63	0.77	0.69	0.59	0.70	0.74	0.53

Non-nucleolar	0.50	200	100	344	0.90	0.91	0.81	0.94	0.93	0.87

^a ^Test set 1: leave-one-out cross-validation test

^b ^Test set 2: independent literature-based test

^c ^Test set 3: SILAC experimental independent test

^d ^These sensitivity measures represent average sensitivity values over ten runs. Their standard deviations were all below 0.03.

^e ^PPV: positive predictive value. These measures represent average PPV values over ten runs. Their standard deviations were all below 0.1.

#### Independent literature test

The classifier was then tested on a literature-based test set which consists of proteins reported to be nucleolar-associated in the literature but that were not used to train the predictor (as described in the Methods). Once again, all classes obtain values well above what you would expect by chance. The overall accuracy of the predictor using the independent literature test is 0.85.

#### SILAC independent test

**Figure 2:**
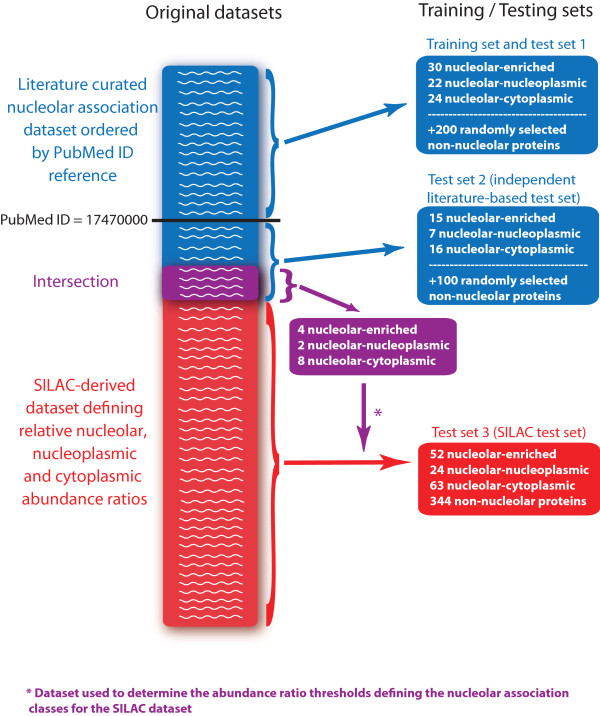
**Generation of the training and testing sets**. Two datasets were used to generate the training and testing sets. A manually curated literature-based nucleolar association dataset (blue list) was used to construct the training set (which is also used in the leave-one-out cross validation test and referred to as the test set 1) and a non-overlapping independent literature-based test set (test set 2). An experimental SILAC dataset (red list) was used to construct the independent SILAC-derived test set (test set 3). The intersection of the manually curated literature dataset (blue list) and the experimental SILAC dataset (red list) is shown in purple and was used to map the SILAC data points to our nucleolar association groups to create the SILAC test set. The generation of the training and testing sets is described in more detail in the Methods section.

The PNAC classifier was further tested with a SILAC (stable isotope labelling with amino acids in cell culture)-derived test set which consists of proteins whose ratio of relative abundance has been measured experimentally over two pairs of cellular compartments: each protein has an associated nucleolar/cytoplasmic abundance ratio and a nucleoplasmic/cytoplasmic abundance ratio (as described in the Methods section and in [12]). While there is no direct relationship between these ratios and our nucleolar association classes, they provide a means to map proteins into these classes. Proteins that belong to both the independent literature test set and the independent SILAC test set were used to determine abundance ratio thresholds defining the nucleolar association classes (as detailed in the Methods and Figure 2). The sensitivity and PPV values for the SILAC test are lower than the equivalent measures for the other tests although they are all significantly higher than what would be expected by chance (i.e. the class priors, see Table 2). The overall accuracy of the SILAC test is 0.74.

There are several reasons why the SILAC test accuracy values are lower than those of the other tests. Firstly, the thresholds used to define the nucleolar association class to which SILAC characterised proteins belong were determined using a very small number of proteins. As shown in Figure 2 (purple box), the intersection between the literature curated dataset (blue list) and the SILAC-derived dataset (red list), which was used to map SILAC experimental ratios into the nucleolar-association classes only consists of fourteen proteins. The thresholds most likely do not perfectly define the nucleolar association groups and will improve when the intersection between the literature-curated dataset and the SILAC-derived datasets increases in size. Secondly, the SILAC test set consists of a larger number of proteins than considered in the other tests (see Table 2, SILAC test set Counts) and is most likely not biased towards proteins that are well annotated, as the much smaller literature datasets would be. As the predictions depend in part on annotations (such as scores generated using the GO and subcellular localisation-derived features), the prediction accuracy will increase as proteins become better annotated. Finally, the SILAC test set is not characterising nucleolar association exactly in the same way as we seek to do here. As such, the two approaches are somewhat complementary in their aims. The SILAC-derived dataset investigates one cell type under normal growth conditions, thus providing a snapshot of the abundance of the proteins in each of the compartments considered under those conditions. In contrast, our method aims to classify human proteins according to their degree of nucleolar association under any possible condition and in all cell types. So while the SILAC test set is useful to increase the coverage of our test sets and investigate the classifier accuracy on proteins that might be highly uncharacterized, the SILAC test set is not a perfect match to test our classifier. This is particularly obvious for proteins that associate with the nucleolus only under specific conditions that were not experimented on in the SILAC-derived dataset. This most notably concerns nucleolar-nucleoplasmic and nucleolar-cytoplasmic proteins.

Table 3 shows examples of disagreements between our classification and the SILAC classification. Disagreements observed between the two methods can be grouped into three general classes:

**Table 3:** Examples of disagreements between SILAC classification and our predictions

Accession	Name	SILAC classification	PNAC classification	Experimental observations from literature
NP_006588	HSPA8	Non-nucleolar (highly cytoplasmic)	Nucleolar-cytoplasmic	Usually cytoplasmic but accumulates in nucleoli after heat-shock [43,44]

NP_001013	RPS19	Non-nucleolar (highly cytoplasmic)	Nucleolar-cytoplasmic	Ribosomal protein which accumulates in the nucleolus [45]

NP_919223	HNRNPA3	Non-nucleolar (mainly nucleoplasmic)	Nucleolar-enriched	The Human Proteome Atlas finds it in the nucleolus, nucleus and cytoplasm [46]

NP_002120	HMGB2	Non-nucleolar (mainly cytoplasmic but also nucleoplasmic)	Nucleolar-nucleoplasmic	The Human Proteome Atlas finds it to be strongly nucleolar [46]

NP_061185	RCC2	Mainly nucleoplasmic	Nucleolar-nucleoplasmic	Annotated in Uniprot as nucleolar, cytoplasmic and centromere

NP_002408	Antigen KI-67	Highly enriched in nucleolus	Nucleolar-nucleoplasmic	Annotated in Uniprot as predominantly perinucleolar in G1 and in later phases predominantly localised in the nuclear matrix [30]

-Proteins that are conditionally localised to the nucleolus: these are proteins that are generally highly non-nucleolar but translocate to the nucleolus under specific conditions. For example, several heat shock proteins including HSPA8 are usually cytoplasmic but are known to translocate to the nucleolus after heat-shock, which was not a condition considered in the SILAC analysis (see Table 3).

-Proteins that are cyclically localised to the nucleolus, often in a cell-cycle manner, for example RCC2 and KI-67 in Table 3.

-Unknown proteins for which little information is available to confirm the true localisation.

In all these cases, a disagreement between the two classification methods warrants further investigation.

### Reliability analysis

**Figure 3:**
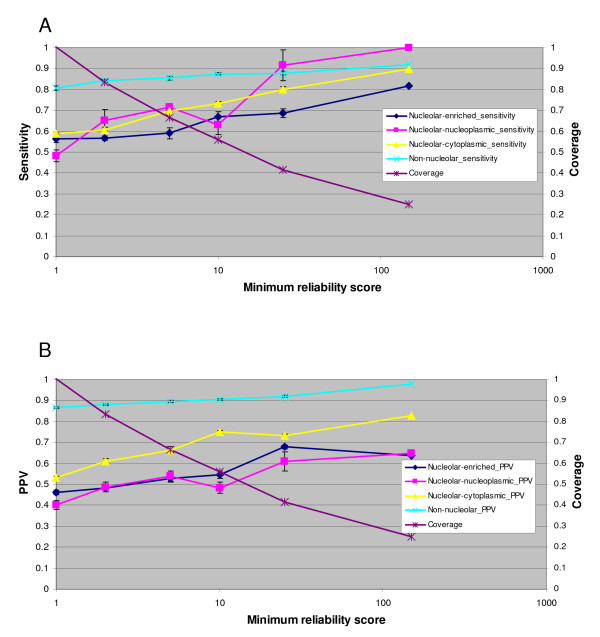
**Reliability analysis**. The sensitivity (panel A) and positive predictive value (PPV; panel B) are plotted as a function of the minimum reliability score for all four classes considered. The error bars represent standard deviation over 10 independent runs.

As described above, PNAC outputs one score per class and proteins are classified as belonging to their highest scoring class. For some proteins, one class clearly scores much higher than the other classes. However, for other proteins, two or more classes have equally high scores indicating that the classifier does not have enough information to make a clear-cut decision. The reliability index measures the fold difference between the highest scoring class and the second-highest scoring class. We investigated whether increases in the reliability index of the classification resulted in increases in the sensitivity and PPV of the classification, using the SILAC-derived independent test-set. Figure 3 shows that as the minimum reliability index increases from 1 to 150, the sensitivity and PPV both increase significantly, providing a means to offer higher quality classifications for a subset of the proteome. The overall accuracy thus goes from 0.74 for the entire test set (minimum reliability index of 1.0) to 0.83 when the minimum reliability index is 10 (which provides a coverage of 56%) and 0.90 when the minimum reliability index is 150 (with a coverage of 25%). The nucleolar association classification and its reliability are provided for all human proteins (as defined by IPI version 3.40 [27]) in Additional File 2.

### Biological process annotations of nucleolar-associated proteins

To characterise the predicted nucleolar-associated proteins, we considered the GO biological process annotations of nucleolar-enriched, nucleolar-nucleoplasmic and nucleolar-cytoplasmic proteins (Table 4). Of the 386 proteins classified as nucleolar-enriched with a reliability index greater than 10, 163 are annotated with RNA metabolic process or any of its children terms including 35 proteins annotated with rRNA processing and 27 annotated with tRNA processing. The second and third most abundant biological process terms for nucleolar-enriched proteins are respectively cellular component organisation with which 81 proteins are annotated and ribosome biogenesis with which 50 proteins are annotated. Taken together, these are the most representative biological process GO terms for nucleolar-enriched proteins and correspond well with the most prominent and well-characterised nucleolar functions.

**Table 4:** Most abundant biological process GO annotations of nucleolar-associated proteins with reliability index above 10

Biological process GO term	**Protein count**^**a**^
**Nucleolar-Enriched**	

RNA metabolic process (GO:0016070)	163
of which rRNA processing (GO:0006364)	35
tRNA processing (GO:0008033)	27
Transcription, DNA-dependent (GO:0006351)	35

Cellular component organization (GO:0016043)	81

Ribosome biogenesis (GO:0042254)	50
of which rRNA processing (GO:0006364)	35

Regulation of biological process (GO:0050789)	45

**Nucleoplasmic-nucleolar**	

Nucleobase, nucleoside, nucleotide and nucleic acid metabolic process (GO:0006139)	118
of which DNA repair (GO:0006281)	52
DNA replication (GO:0006260)	43

Regulation of biological process (GO:0050789)	107
of which Regulation of transcription, DNA-dependent (GO:0006355)	35
Signal transduction (GO:0007165)	36

Cellular component organization (GO:0016043)	92
of which Chromosome organisation (GO:0051276)	68

Cell cycle (GO:0007049)	89

Multicellular organismal development (GO:0007275)	40

Cell proliferation (GO:0008283)	39

Cell death (GO:0008219)	27

**Nucleolar-cytoplasmic**	

Protein metabolic process (GO:0019538)	127
of which Translation (GO:0006412)	106

Nucleobase, nucleoside, nucleotide and nucleic acid metabolic process (GO:0006139)	33

Regulation of biological process (GO:0050789)	27
of which Signal transduction (GO:0007165)	10

Cellular component organisation (GO:0016043)	18
of which Organelle organisation (GO:0006996)	11

^a ^count includes proteins annotated with child terms

In contrast, the 513 nucleolar-nucleoplasmic and 469 nucleolar-cytoplasmic classified proteins with reliability index above 10 are annotated with a wide variety of different terms. As shown in Table 4, the biological process term annotating the largest number of nucleolar-nucleoplasmic proteins is nucleobase, nucleoside, nucleotide and nucleic acid metabolic process which includes 52 proteins involved in DNA repair and 43 in DNA replication. Other biological process terms highly populated with nucleolar-nucleoplasmic proteins include cell cycle, chromosome organisation, regulation of transcription, DNA-dependent and signal transduction, in agreement with more recently described nucleolar-associated functions. Unsurprisingly, in the case of nucleolar-cytoplasmic proteins, the most predominant biological process is protein metabolic process, which includes 106 proteins annotated with the term translation.

### Analysis of the evolution of nucleolar-associated proteins

**Figure 4:**
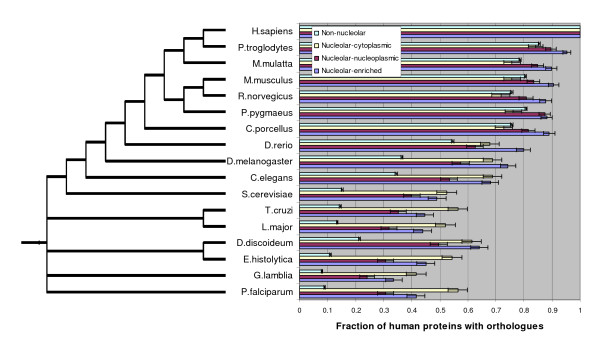
**Conservation analysis of nucleolar-associated proteins**. For each nucleolar-association group and the non-nucleolar group, the fraction of proteins with orthologues in a given eukaryotic organism was examined. Only proteins with reliability index above 10 were considered. The error bars represent standard deviation as estimated using a bootstrap procedure.

The different nucleolar association groups investigated here are thus composed of proteins involved in a wide variety of biological processes. The nucleolus is traditionally associated with highly conserved protein families and core cellular functions. However, numerous recent studies also involve the nucleolus in other diverse cellular processes. In an effort to characterise the nucleolar protein content evolutionarily, we investigated the fraction of human proteins of each nucleolar association group that have orthology (as defined by InParanoid [28]) to proteins in other eukaryotic organisms. To ensure that we are dealing with highly accurate nucleolar association predictions, we only consider the proteins with a nucleolar association reliability index classification greater than 10. As shown in Figure 4, the three different nucleolar association groups (nucleolar-enriched, nucleolar-nucleoplasmic and nucleolar-cytoplasmic) display different conservation patterns. However, the most striking feature of this analysis is that in all non-mammalian organisms considered, the non-nucleolar group displays a much lower degree of conservation than any of the nucleolar association groups, and the gap between the non-nucleolar and nucleolar groups greatly increases with evolutionary distance. Thus proteins associated with all aspects of nucleolar functions are much better conserved throughout eukaryotic evolution than proteins that never associate with the nucleolus.

The nucleolar-cytoplasmic group, which consists largely of proteins involved in translation (see Table 4), has a very high fraction of human proteins with orthologues in other organisms. This is consistent with the slow evolution and high conservation that has previously been shown for many proteins of this group [29]. Of the 202 nucleolar-cytoplasmic human proteins considered, the fraction with orthology to the non-mammalian organisms considered ranges between 42% (for *Giardia lamblia*) to 69% (for *Drosophila melanogaster*). In contrast, of the 12725 human proteins considered for the non-nucleolar group, between 8% (for *Giardia lamblia*) and 55% (for *Danio rerio*) of non-nucleolar proteins have orthologues in the non-mammalian eukaryotic organisms considered. Nucleolar-enriched and nucleolar-nucleoplasmic proteins are often not as well conserved as nucleolar-cytoplasmic proteins especially in the most distant non-mammalian eukaryotic organisms considered but are significantly more conserved than non-nucleolar proteins.

In mammals, the nucleolar-enriched and nucleolar-nucleoplasmic groups have the highest fraction of orthologues with between 81% (in the case of human nucleolar-nucleoplasmic proteins with orthology to *Rattus norvegicus*) and 95% (in the case of human nucleolar-enriched proteins with orthology to *Pan troglodytes*) of their proteins having mammalian orthologues.

Thus many of the central processes carried out, at least in part, by nucleoli exist in all eukaryotes considered and, compared to non-nucleolar proteins, a much higher proportion of nucleolar-associated proteins are conserved amongst eukaryotic organisms.

## Conclusions

In an effort to predict and describe the nucleolar proteome, we investigated the integration of various gene and protein features and annotations in a naïve Bayesian framework. To help differentiate between core-nucleolar proteins and proteins that associate with the nucleolus temporarily but also function in other compartments, the training set was subdivided into four groups: nucleolar-enriched, nucleolar-nucleoplasmic, nucleolar-cytoplasmic and non-nucleolar proteins. This classification scheme provides information regarding the nucleolar-association potential of all human proteins in a manner that is neither cell-type, nor condition-specific. An analysis of our proteome-wide nucleolar-association predictions reveals that these groups display widely varying evolutionary characteristics and biological process signatures. This classification provides a clearer picture of the protein content of the nucleolus as well as its numerous and central roles in the cell and its interaction with other subcellular compartments.

## Methods

### Datasets

#### Training sets

Human proteins experimentally detected in the nucleolus were manually curated from the literature and inserted into one of three groups depending on their degree of association with the nucleolus:

-The nucleolar-enriched class consists of proteins found to be predominantly nucleolar in all cell types and conditions considered (for examples, see Additional File 1). Thirty proteins are included in the nucleolar-enriched training set.

-The nucleolar-nucleoplasmic class is composed of nuclear proteins that are identified in several nuclear regions including the nucleolus. This includes proteins that cycle between the nucleolus and other nuclear regions and proteins that localise primarily to non-nucleolar nuclear regions but relocate to the nucleolus under specific conditions (for examples, see Additional File 1). Twenty-two proteins form the nucleolar-nucleoplasmic training set.

-The nucleolar-cytoplasmic class consists of proteins that are mainly cytoplasmic but have been detected in the nucleolus. This includes proteins that cycle between the cytoplasm and the nucleolus, cytoplasmic proteins that are assembled into larger complexes in the nucleolus as well as cytoplasmic proteins that are detected in the nucleolus under specific conditions (for examples, see Additional File 1). The nucleolar-cytoplasmic training set consists of twenty-four proteins.

The non-nucleolar group was generated by randomly choosing proteins from IPI release 3.40 [27] that are not annotated as being nucleolar in UniProt [30] or HPRD [31].

The PNAC classifier was trained on 200 randomly chosen non-nucleolar proteins as well as all proteins from the manually curated nucleolar datasets (listed in Additional File 1) whose earliest nucleolar association literature reference (according to Additional File 1) has a PubMed ID smaller than 17470000 (which corresponds approximately to the first half of 2007). While most of the nucleolar association literature references considered here describe work performed in a small-scale manner (see references in Additional File 1), three large scale projects were included [9-11], mainly to ensure the presence of ribosomal proteins in the nucleolar-cytoplasmic dataset. Two of these projects [10,11] were considered to generate the training set while the third [9] was considered to generate test set 2 (see below), even though its pubmed ID is below 1747000. The training set generation scheme is depicted in Figure 2.

#### Testing sets

The accuracy of the PNAC classifier was measured using three different test sets (Figure 2):

##### Test set 1

A leave-one-out cross-validation test in which one training set protein is set aside for testing purposes and the classifier is trained on all the remaining training set proteins. This is repeated for all training set proteins.

##### Test set 2

An independent, literature-based test in which the classifier is trained on all training set proteins and then tested on the remaining literature-curated proteins whose earliest PubMed ID nucleolar association reference (according to Additional File 1) is greater than 17470000 as illustrated in Figure 2. As explained above (Training set section), some ribosomal proteins reported to be nucleolar-associated in [9] were also included in this test set even though its pubmed ID is below 17470000, to ensure the presence of ribosomal proteins in test set 2. These ribosomal proteins were not included in the training set.

##### Test set 3

An independent experimental dataset generated using SILAC (stable isotope labelling with amino acids in cell culture). This dataset consists of a list of proteins whose relative abundance has been measured by harvesting nucleolar, nucleoplasmic and cytoplasmic cellular extracts each grown in the presence of amino acids labelled with different isotopes and then by pooling together the different fractions and analysing them by mass spectrometry [12]. Each protein is thus assigned two ratios: a nucleolar versus cytoplasmic ratio and a nucleoplasmic versus cytoplasmic ratio which define the relative abundance of the protein in these three compartments. The SILAC independent protein dataset was partitioned into five groups (nucleolar-enriched, nucleolar-nucleoplasmic, nucleolar-cytoplasmic, non-nucleolar, undefined) depending on their nucleolar versus cytoplasmic and nucleoplasmic versus cytoplasmic ratios (see Additional File 3). The thresholds used to define the five groups were determined manually by careful consideration of the proteins that are both in the independent literature based test set (test set 2) and in the independent SILAC set (test set 3) as depicted in Figure 2. These proteins that form the intersection of test set 2 and test set 3 are listed in Additional File 4 which also defines the thresholds used to decide to which nucleolar association group different SILAC characterised proteins belong. The fifth group (undefined group) corresponds to all proteins that do not fall into any of the four previously defined groups (their ratios are too different from the ratios of the proteins used to determine the thresholds). The accuracy results shown in Table 2 for the independent SILAC test exclude all proteins that were trained on or used to determine the SILAC thresholds to define the SILAC groups.

### Redundancy filtering

Redundancy in the training and test sets was eliminated by ensuring that all proteins are less than 25% identical over their entire sequence to any other protein in the datasets.

### Features

PNAC considers five distinct features to classify proteins according to their degree of nucleolar association:

#### 1) Amino acid frequency

The frequency of most individual amino acids does not differ significantly between the proteins of the different nucleolar-association training set groups. However, in the case of serine, leucine, isoleucine and lysine, there are significant differences in their frequency between the different nucleolar-association groups. The frequency of each of these amino acids was measured for each protein considered and then the frequencies were grouped into five bins (four for lysine) using thresholds determined empirically.

#### 2) Presence of targeting motifs and transmembrane domains (TMDs)

The presence of signal peptides and number of TMDs were predicted for each protein by Phobius [32]. In addition to that, for each protein, the presence of mitochondrial targeting peptides and of nucleolar localisation sequences (NoLSs) were predicted respectively by TargetP [33] and NoD [34]. Other targeting motifs were also considered but did not offer the same predictive capability as the chosen targeting motifs.

The results of these predictions were used to define three scores that characterise targeting motifs in a protein:

Mitochondrial score s_M _= 1 if TargetP predicts a mitochondrial targeting peptide, s_M _= 0 otherwise.

Secretory-membrane score s_S _= 1 if Phobius predicts a signal peptide or at least one TMD, s_S _= 0 otherwise.

NoLS score s_N _= 2 if the maximum NoLS score output by NoD is > = 0.9.

s_N _= 1 if the maximum NoLS score output by NoD is between 0.8 and 0.9.

s_N _= 0 if the maximum NoLS score output by NoD is <0.8.

The cyto score s_C _is defined based on s_M _and s_S _such that s_C _= 2 if s_S _= 1 regardless of s_M_, s_C _= 1 if s_M _= 1 and s_S _= 0 and s_C _= 0 if s_M _= 0 and s_S _= 0.

These s_C _and s_N _scores were grouped into nine bins representing all possible combinations of their states and their distribution is plotted for each class in Additional File 5.

#### 3) Gene co-expression with nucleolar-cytoplasmic group

The average Pearson correlation of co-expression between the query protein and proteins in the nucleolar-cytoplasmic training group was calculated for all proteins considered, using expression profiles from 79 physiologically normal tissues [35]. The correlation values were then grouped into four bins containing roughly (within 20%) the same number of proteins using thresholds determined empirically. Gene co-expression correlations with the other nucleolar association groups were also considered but these correlation values were not found to be predictive of nucleolar association. Thus only gene co-expression correlation with the nucleolar-cytoplasmic group was used.

#### 4) Gene Ontology (GO) annotations

Biological process and molecular function GO annotations [36] were downloaded for all proteins considered. For a given GO term *t *annotating a query protein, the ratio of the number of proteins annotated with term *t *in a given nucleolar association class versus the number of proteins in the entire human proteome annotated with term *t *was calculated for each nucleolar association class (i.e. for the nucleolar-enriched, nucleolar-nucleoplasmic and nucleolar-cytoplasmic classes). For query proteins annotated with more than one term, these ratios were averaged over all of their annotating terms for each nucleolar association class c, producing one such GOscore *g*_*c *_per nucleolar association class, for a given protein:

gc=∑t∈Tpnctnt|Tp|

where T_p _is the set of all terms that are associated with protein p, n_ct _is the number of proteins of nucleolar association class c that are annotated with term t and n_t _is the total number of human proteins annotated with term t.

These ratios are then grouped into one of four bins, depending on which nucleolar association GOscore g_c _is highest: proteins whose nucleolar-enriched GOscore was highest, proteins whose nucleolar-nucleoplasmic GOscore is highest, proteins whose nucleolar-cytoplasmic GOscore is highest and those whose GOscores all fall below a threshold set to 0.003.

#### 5) Subcellular localisation annotations of interactors

A nucleolar proximity score was calculated for all interactors of the query protein using HPRD [31] and Uniprot [30] subcellular localisation annotations. To do so, protein localisation annotations were grouped into four cellular regions which were each assigned a nucleolar proximity distance:

• 0.0 for the nucleolus

• 1.2 for the nucleoplasm, the nuclear speckles, the nuclear pore and the nuclear envelop

• 3.0 for the cytosol, cytoplasm, any of the cytoplasmic organelles, the plasma membrane and extracellular region

• 0.8 for the 'nuclear' annotation as this does not distinguish between nucleolar and nuclear non-nucleolar proteins.

Given these distances, each protein p was attributed an average nucleolar proximity score NPI for all its interactors as follows:

$$NPI_{p}=\frac{\sum_{i\in I}NP_{i}}{\left|I\right|}$$

where

$$NP_{i}=\frac{\sum_{r\in R_{i}}D_{r}}{\left|R_{i}\right|}$$

and where I is the set of all interactors of the query protein, D_r _is the distance between the nucleolus and cellular region r and R_i _is the set of cellular regions to which protein i (the interactor) localises.

The protein interactions considered include all protein pairs predicted by the human protein-protein interaction predictor PIPs to interact with a posterior odds ratio above 4 [37,38] as well as protein pairs annotated as interacting in HPRD [31] and IntAct [39].

The NPI_p _scores were grouped into 5 bins according to manually selected thresholds that were optimised to minimise the average class error in the leave-one-out cross-validation test. For all tests, care was taken to remove all interactors of the current test protein from consideration in calculating the NPI score of all training proteins.

Protein interaction data have been considered previously in the prediction of protein subcellular localisation, including for whole-cell protein localisation prediction [16,40,41] as well as by most of the nucleolar binary predictors [23,24,26].

### Learning method

Semi-naïve Bayes classifiers were used to score the likelihood of localisation to the four classes considered, based on the features described above. This learning method was chosen because of its transparency and ease of integration of highly heterogeneous data. The method was trained by counting the number of proteins from the different training classes that fall into each bin. Pseudocounts of 0.1 were added to all bins to ensure that no feature state would obtain infinite scores. The bin counts for each class were then divided by the total number of proteins that fall in the bin, regardless of their class, thus obtaining a conditional probability table for each feature considered. The five different features described above were considered independent and thus the final score for each class is calculated as the product of the initial class prior by the scores calculated for the individual features. The initial class priors were chosen to minimise the average class error in the leave-one-out cross-validation test and are set to 0.2 for the nucleolar-enriched class, 0.15 for the nucleolar-nucleoplasmic class, 0.15 for the nucleolar-cytoplasmic class and 0.5 for the non-nucleolar class. Proteins are labelled as belonging to their highest scoring class.

### Measures of accuracy

The classifier's accuracy for the three tests described above is measured by calculating the sensitivity and positive predictive value (PPV) for each nucleolar-association class:

$$\begin{array}{l} Sensitivity=\frac{TP}{TP+FN}\\ PPV=\frac{TP}{TP+FP} \end{array}$$

The sensitivity measures the fraction of true positives (TP) amongst all the proteins annotated as being positives for this class in this particular test. The PPV measures the fraction of true positives amongst all the proteins predicted to be positive for this class. FN and FP represent respectively the false negative and false positive counts.

The overall accuracy for a given test is defined as the number of well-predicted proteins divided by the total number of proteins in the test set.

All measures of accuracy presented in Table 2 represent averages over ten runs. All runs are individually trained on the same nucleolar-enriched, nucleolar-nucleoplasmic and nucleolar-cytoplasmic sets but differ in their non-nucleolar sets, which are randomly generated as described in the Methods Dataset section.

### Reliability Index

The reliability index (RI) of the classification is calculated as the ratio of the score of the highest scoring class divided by the score of the second highest scoring class.

### GO biological process annotations of predicted nucleolar-associated proteins

Biological process GO annotations [36] were downloaded for nucleolar-enriched, nucleolar-nucleoplasmic and nucleolar-cytoplasmic classified human proteins with reliability index greater than 10.0. Proteins can be annotated with more than one term.

### Evolutionary analysis

For each nucleolar-association group, the number of proteins with orthologues in a given organism (as predicted by InParanoid7 [28]) was counted and compared to the total number of proteins of this group that are considered by InParanoid7. The standard deviation of these measures was estimated by a bootstrap procedure (using 1000000 bootstrap datasets derived using the inParanoid orthology predictions for each nucleolar-association group).

## Authors' contributions

MSS conceived and designed the study, curated the literature-derived datasets, created the classifier, carried out the analysis and drafted the manuscript. FMB generated the SILAC dataset and participated in the analysis of the predictions. AIL contributed to the analysis and participated in drafting the manuscript. GJB participated in the design of the study, its analysis and helped to draft the manuscript. All authors read and approved the final manuscript.

## Supplementary Material

Additional file 1 **Literature curated nucleolar-association lists**. This file lists nucleolar-enriched, nucleolar-nucleoplasmic and nucleolar-cytoplasmic proteins and the literature references in which their association with the nucleolus was experimentally investigated. Click here for file

Additional file 2 **Nucleolar association classification for all human proteins**. The PNAC prediction of nucleolar association is given for all human proteins from IPI release 3.40. Click here for file

Additional file 3 **SILAC-derived dataset**. This list displays the SILAC-derived dataset protein identifiers and abundance ratios. Click here for file

Additional file 4 **Description of discretization of SILAC abundance ratios into PNAC categories**. This file lists the SILAC proteins used to define the SILAC ratio thresholds and displays the thresholds. Click here for file

Additional file 5 **Distribution of protein targeting motif scores per class**. This file displays a plot of the distribution of scores for the targeting motif module. Click here for file
