# Supplementary material for: PNAC: a protein nucleolar association classifier
Source: BMC Genomics. 2011 Jan 27;12:74. doi: 10.1186/1471-2164-12-74 (PMC3038921; doi:10.1186/1471-2164-12-74)
Supplement: Additional file 1 — Literature curated nucleolar-association lists. This file lists nucleolar-enriched, nucleolar-nucleoplasmic and nucleolar-cytoplasmic proteins and the literature references in which their association with the nucleolus was experimentally investigated. [file 1471-2164-12-74-S1.PDF]

Additional Table I: Proteins belonging to the nucleolar-only class manually curated from the literature.

RefSeq	Name	References
NP_055118	pescadillo homolog 1, containing BRCT domain	[1]
NP_060014	transcription factor NRF	[2]
O15446-2	DNA-directed RNA polymerase I subunit RPA34	[3]
NP_056139	ribosomal RNA-processing protein 8	[4]
NP_004695	U3 small nucleolar interacting protein 2	[5]
NP_006744	surfeit 6	[6]
NP_055318	down-regulated in metastasis	[7]
NP_055701	ribosome biogenesis protein NSA2 homolog	[8]
NP_055048	upstream binding transcription factor, RNA polymerase I isoform a	[9,10]
NP_001427	fibrillarin	[11]
NP_001091059<br>NP_006405<br>NP_892117	ribonuclease P/MRP 38 subunit	[12]
NP_000347	treacle protein isoform b	[13,14]
NP_056016	block of proliferation 1	[15]
NP_055791	programmed cell death 11	[16,17]
NP_055984	ribosome biogenesis regulatory protein homolog	[18]
NP_734467	selenoprotein H	[19]
NP_777611	family with sequence similarity 9, member A	[20]
NP_001036100	neuroguidin	[21]
NP_115979	JHDM1B	[22]
NP_219484	IMP4, U3 small nucleolar ribonucleoprotein, homolog	[23]
NP_057409	zinc finger protein 274 isoform a	[24]
NP_055335	MYB binding protein 1a isoform 2	[25,26]
NP_060726	WD repeat domain 12 protein	[27]
NP_078938	N-acetyltransferase 10 isoform a	[28]
NP_056485	SMT3IP1	[29]
NP_075068	nucleolar protein family 6 alpha isoform	[30]
NP_115570	BXDC1	[31]
NP_004719	DEAD (Asp-Glu-Ala-Asp) box polypeptide 21	[32,33]
NP_060755	U3 snoRNP protein 3 homolog	[34]
NP_060547	RBM28	[35]
NP_060308	nucleolar protein family A, member 2 isoform a	[36]
NP_060286	Ly1 antibody reactive homolog	[37]
NP_061955	ATP-dependent RNA helicase DDX56	[38]
NP_057267	mRNA turnover protein 4 homolog	[39]
NP_057251	NOL7	[40]
NP_612409	nucleolar protein with MIF4G domain 1 (NOM1)	[41,42]
Q8IUF8-3	MYC induced nuclear antigen	[43]
NP_004732	NOPP140	[44]
NP_078920	NO66	[45]
NP_076977	DEAD (Asp-Glu-Ala-Asp) box polypeptide 54 isoform 2	[46]
NP_001030008	RAD52 motif-containing protein 1 isoform 2	[47]
NP_061940	guanine nucleotide binding protein-like 3	[48]
NP_006383	nucleolar protein 5A	[49]
NP_057722	ADP-ribosylation factor-like protein	[50]
NP_006815	EBNA1 binding protein2 isoform 2	[31]

Additional Table II: Proteins belonging to the nucleolar-nucleoplasmic class manually curated from the literature.

RefSeq	Name	Comments	References
NP_002476	nibrin	nucleoplasmic, nucleolar after DNA damage	[51-53]
NP_004251	RECQL4	nucleoplasmic and faintly nucleolar but in presence of hydrogen peroxide relocalizes to nucleoli	[54]
NP_000312	Rb1	nucleoplasmic but can be sequestered in the nucleolus	[55]
NP_066964	Ku80, XRCC5	nucleolar and nucleoplasmic depending on cell cycle phase	[56]
NP_005905 NP_877423	MCM4	nucleoplasmic and nucleolar	[57]
NP_006221	polymerase (DNA directed), delta 2, regulatory subunit	nucleoplasmic but relocalizes to the nucleolus in the presence of Werner (WRN) protein	[58]
NP_059523	telomeric repeat-binding factor 1 isoform 1	present in nucleoplasm and nucleolus	[59]
NP_778224	histone H4	present in promoters of rDNA which localize to the nucleolus	[60]
NP_001001998	exosome component 10	mostly nucleolar but also nucleoplasmic	[61]
NP_000544	WRN	its nucleolar localization seems to depend on the cell type, in HeLa cells, it is only nucleolar whereas in other cell types, it is more nucleoplasmic	[62]
NP_005672	TBP-associated factor 1A isoform 1	nuclear and nucleolar	[63]
NP_003484	histone H3	present in promoters of rDNA which localize to the nucleolus	[60,64]
NP_778243	aprataxin isoform a	nucleoplasmic and nucleolar, has acitivities in both compartments	[65]
NP_001015877 NP_115834	PHF6	nucleolar and strongly nucleoplasmic	[66]
NP_478102	ARF	nucleolar and nucleoplasmic	[67]
NP_006017	histone H1X	nucleolar and nucleoplasmic, depending on phase of cell cycle	[68]
NP_116219	cirhin	mainly nucleolar but also nucleoplasmic	[69,70]
NP_001012300	p78/MCRS1	nucleoplasm and nucleolus	[71,72]
NP_536349	SP110	nucleolar and nucleoplasmic	[73]
NP_001093882	drosha isoform 2	nucleoplasmic but also nucleolar in S phase	[74]
NP_937862	ING1	nucleoplasmic but translocates to nucleoli after UV irradiation	[75]
NP_057122	SBDS	nucleoplasmic and nucleolar	[76]
NP_056422	SRAG	nucleoplasmic and sometimes nucleolar	[77]
NP_073604	apoptosis-enhancing nuclease	translocates from nucleolus to nucleoplasm upon apoptosis induction	[78]
NP_006616	SRp38	nucleoplasmic and mostly perinucleolar	[79]
NP_002383	MDM2	nucleoplasmic and nucleolar when sequestered by Arf	[80]
NP_004866	DNA-directed RNA polymerases I and III subunit	nucleolar and nucleoplasmic	[81]
NP_056075	SIN3B	nucleoplasmic and nucleolar	[82]
NP_005863	BRCA2	nuclear and detected in nucleolus in large-scale study	[83,84]

Additional Table III: Proteins belonging to the nucleolar-cytoplasmic class manually curated from the literature.

RefSeq	Name	Comments	References
NP_003895	ZPR1	Cytoplasmic but accumulates in the nucleolus of mitogen activated cells	[85]
NP_001034800 NP_001034801 NP_127491	DEDD	Cytoplasmic, translocates to nucleus and nucleolus upon stimulation of CD95	[86,87]
NP_008878	SRP72	Part of cytoplasmic complex assembled in nucleolus	[88]
NP_002573	DAN, PARN	Cytoplasmic but also nucleoplasmic and nucleolar	[89]
NP_000993 NP_444505	Ribosomal protein P0	Cytoplasmic and nucleolar	[90]
NP_003126	SRP19	Part of cytoplasmic complex assembled in nucleolus	[88]
NP_005238	FGF3	Nuclear/nucleolar and also secreted	[91]
NP_002811 NP_945315	PTHLH	Secreted but can localize to the nucleolus	[92]
NP_000976 NP_001030178	ribosomal protein L17	Part of the ribosome which functions in the cytoplasm and is assembled in the nucleolus. Detected in large-scale study of nucleolus.	[93,94]
NP_000968 NP_150254	ribosomal protein L13	Part of the ribosome which functions in the cytoplasm and is assembled in the nucleolus. Detected in large-scale study of nucleolus.	[93]
NP_000967	ribosomal protein L12	Part of the ribosome which functions in the cytoplasm and is assembled in the nucleolus. Detected in large-scale study of nucleolus.	[93]
NP_005535	insulin receptor substrate 1	Localizes to cytosol but under certain conditions goes to nucleus and nucleolus	[95,96]
NP_000959	ribosomal protein L4	Part of the ribosome which functions in the cytoplasm and is assembled in the nucleolus. Detected in large-scale study of nucleolus.	[93]
NP_055555	eukaryotic initiation factor 4A-III	Nucleocytoplasmic shuttling protein, travels to the cytoplasm bound to mRNA as part of the exon junction complex. Relocalizes to the nucleolus under stress conditions	[97-99]
NP_036555	ribosomal protein L13a	Part of the ribosome which functions in the cytoplasm and is assembled in the nucleolus. Detected in large-scale study of nucleolus.	[93]
NP_002641	pi4K230	Cytoplasmic and membrane bound but also detected in nucleolus	[100]
NP_000981	ribosomal protein L27a	Part of the ribosome which functions in the cytoplasm and is assembled in the nucleolus. Detected in large-scale study of nucleolus.	[93]
NP_000960	ribo prot L5	Part of the ribosome which functions in the cytoplasm and is assembled in the nucleolus.	[101]
NP_001000	ribosomal protein S5	Part of the ribosome which functions in the cytoplasm and is assembled in the nucleolus. Detected in large-scale study of nucleolus.	[93]
NP_001002	ribo prot S7	Part of the ribosome which functions in the cytoplasm and is assembled in the nucleolus.	[101]
NP_001003	ribosomal protein S8	Part of the ribosome which functions in the cytoplasm and is assembled in the nucleolus. Detected in large-scale study of nucleolus.	[93]
NP_072045	ribosomal protein S18	Part of the ribosome which functions in the cytoplasm and is assembled in the nucleolus. Detected in large-scale study of nucleolus.	[93]
NP_000975 NP_001087227	ribosomal protein L23a	Part of the ribosome which functions in the cytoplasm and is assembled in the nucleolus. Detected in large-scale study of nucleolus.	[94,101]

NP_000969	ribo prot L23	Part of the ribosome which functions in the cytoplasm and is assembled in the nucleolus. Interacts with MDM2.	[102]
NP_000984	ribosomal protein L31 isoform 1	Part of the ribosome which functions in the cytoplasm and is assembled in the nucleolus. Detected in large-scale study of nucleolus.	[94]
NP_000964 NP_150644	ribosomal protein L8	Part of the ribosome which functions in the cytoplasm and is assembled in the nucleolus. Detected in large-scale study of nucleolus.	[93,94]
NP_000961 NP_001019833	ribosomal protein L6	Part of the ribosome which functions in the cytoplasm and is assembled in the nucleolus. Detected in large-scale study of nucleolus.	[93]
NP_003248	tight junction protein 1 isoform a	Localized to intracellular junctions but also detected in nucleoli	[103]
NP_005029	PPID	Cytoplasmic and nucleolar	[104]
NP_036350	ILF3	Nucleolar and cytoplasmic	[84,105]
NP_001025482	ceramide kinase-like protein isoform b	Mainly cytoplasmic but also localizes to the nucleolus	[106-108]
NP_064709	CIAPIN1	Cytoplasmic and nuclear and accumulates in nucleolus, might undergo a cytoplasm-nucleus-nucleolus translocation	[109]
NP_848927	metadherin	In ER and nuclear envelop but also in nucleolus	[110]
NP_055902	spartin	Mitochondrial, ER and nucleolar	[111]
NP_055045	SRP68	Part of cytoplasmic complex assembled in nucleolus	[88]
NP_001073936	myosin-Vb	Cytoplasmic but also nucleolar	[112]
NP_001030168 NP_003964	ribosomal protein L14	Part of the ribosome which functions in the cytoplasm and is assembled in the nucleolus. Detected in large-scale study of nucleolus.	[93]
NP_001997	fibroblast growth factor 2	Secreted but also detected in nucleolus	[113-116]
NP_001007	ribosomal protein S12	Part of the ribosome which functions in the cytoplasm and is assembled in the nucleolus. Detected in large-scale study of nucleolus.	[93]
NP_060239	G2E3	Cycles between nucleus and cytoplasm but accumulates in nucleoli after DNA damage	[117]

References

1. Kinoshita Y, Jarell AD, Flaman JM, Foltz G, Schuster J, et al. (2001) Pescadillo, a novel cell cycle regulatory protein abnormally expressed in malignant cells. J Biol Chem 276: 6656-6665.
2. Niedick I, Froese N, Oumard A, Mueller PP, Nourbakhsh M, et al. (2004) Nucleolar localization and mobility analysis of the NF-kappaB repressing factor NRF. J Cell Sci 117: 3447-3458.
3. Whitehead CM, Winkfein RJ, Fritzler MJ, Rattner JB (1997) ASE-1: a novel protein of the fibrillar centres of the nucleolus and nucleolus organizer region of mitotic chromosomes. Chromosoma 106: 493-502.
4. Murayama A, Ohmori K, Fujimura A, Minami H, Yasuzawa-Tanaka K, et al. (2008) Epigenetic control of rDNA loci in response to intracellular energy status. Cell 133: 627-639.
5. Pluk H, Soffner J, Luhrmann R, van Venrooij WJ (1998) cDNA cloning and characterization of the human U3 small nucleolar ribonucleoprotein complex-associated 55-kilodalton protein. Mol Cell Biol 18: 488-498.
6. Magoulas C, Zatsepina OV, Jordan PW, Jordan EG, Fried M (1998) The SURF-6 protein is a component of the nucleolar matrix and has a high binding capacity for nucleic acids in vitro. Eur J Cell Biol 75: 174-183.
7. Liu J, Du X, Ke Y (2006) Mapping nucleolar localization sequences of 1A6/DRIM. FEBS Lett 580: 1405-1410.
8. Zhang H, Ma X, Shi T, Song Q, Zhao H, et al. (2010) NSA2, a novel nucleolus protein regulates cell proliferation and cell cycle. Biochem Biophys Res Commun 391: 651-658.
9. Voit R, Hoffmann M, Grummt I (1999) Phosphorylation by G1-specific cdk-cyclin complexes activates the nucleolar transcription factor UBF. EMBO J 18: 1891-1899.
10. Voit R, Kuhn A, Sander EE, Grummt I (1995) Activation of mammalian ribosomal gene transcription requires phosphorylation of the nucleolar transcription factor UBF. Nucleic Acids Res 23: 2593-2599.
11. Ochs RL, Lischwe MA, Spohn WH, Busch H (1985) Fibrillarin: a new protein of the nucleolus identified by autoimmune sera. Biol Cell 54: 123-133.
12. Jarrous N, Wolenski JS, Wesolowski D, Lee C, Altman S (1999) Localization in the nucleolus and coiled bodies of protein subunits of the ribonucleoprotein ribonuclease P. J Cell Biol 146: 559-572.
13. Fujioka H, Ariga T, Horiuchi K, Ishikiriyama S, Oyama K, et al. (2008) Detection of a novel silent deletion, a missense mutation and a nonsense mutation in TCOF1. Pediatr Int 50: 806-809.
14. Winokur ST, Shiang R (1998) The Treacher Collins syndrome (TCOF1) gene product, treacle, is targeted to the nucleolus by signals in its C-terminus. Hum Mol Genet 7: 1947-1952.
15. Strezoska Z, Pestov DG, Lau LF (2002) Functional inactivation of the mouse nucleolar protein Bop1 inhibits multiple steps in pre-rRNA processing and blocks cell cycle progression. J Biol Chem 277: 29617-29625.
16. Sweet T, Khalili K, Sawaya BE, Amini S (2003) Identification of a novel protein from glial cells based on its ability to interact with NF-kappaB subunits. J Cell Biochem 90: 884-891.
17. Sweet T, Yen W, Khalili K, Amini S (2008) Evidence for involvement of NFBP in processing of ribosomal RNA. J Cell Physiol 214: 381-388.
18. Gambe AE, Matsunaga S, Takata H, Ono-Maniwa R, Baba A, et al. (2009) A nucleolar protein RRS1 contributes to chromosome congression. FEBS Lett 583: 1951-1956.
19. Novoselov SV, Kryukov GV, Xu XM, Carlson BA, Hatfield DL, et al. (2007) Selenoprotein H is a nucleolar thioredoxin-like protein with a unique expression pattern. J Biol Chem 282: 11960-11968.
20. Martinez-Garay I, Jablonka S, Sutajova M, Steuernagel P, Gal A, et al. (2002) A new gene family (FAM9) of low-copy repeats in Xp22.3 expressed exclusively in testis: implications for recombinations in this region. Genomics 80: 259-267.
21. Sihn CR, Lee YS, Jeong JS, Park K, Kim SH (2008) CANu1, a novel nucleolar protein, accumulated on centromere in response to DNA damage. Genes Cells 13: 787-796.

22. Frescas D, Guardavaccaro D, Bassermann F, Koyama-Nasu R, Pagano M (2007) JHDM1B/FBXL10 is a nucleolar protein that represses transcription of ribosomal RNA genes. Nature 450: 309-313.
23. Leary DJ, Terns MP, Huang S (2004) Components of U3 snoRNA-containing complexes shuttle between nuclei and the cytoplasm and differentially localize in nucleoli: implications for assembly and function. Mol Biol Cell 15: 281-293.
24. Yano K, Ueki N, Oda T, Seki N, Masuho Y, et al. (2000) Identification and characterization of human ZNF274 cDNA, which encodes a novel kruppel-type zinc-finger protein having nucleolar targeting ability. Genomics 65: 75-80.
25. Tavner FJ, Simpson R, Tashiro S, Favier D, Jenkins NA, et al. (1998) Molecular cloning reveals that the p160 Myb-binding protein is a novel, predominantly nucleolar protein which may play a role in transactivation by Myb. Mol Cell Biol 18: 989-1002.
26. Yamauchi T, Keough RA, Gonda TJ, Ishii S (2008) Ribosomal stress induces processing of Mybbp1a and its translocation from the nucleolus to the nucleoplasm. Genes Cells 13: 27-39.
27. Holzel M, Rohrmoser M, Schlee M, Grimm T, Harasim T, et al. (2005) Mammalian WDR12 is a novel member of the Pes1-Bop1 complex and is required for ribosome biogenesis and cell proliferation. J Cell Biol 170: 367-378.
28. Shen Q, Zheng X, McNutt MA, Guang L, Sun Y, et al. (2009) NAT10, a nucleolar protein, localizes to the midbody and regulates cytokinesis and acetylation of microtubules. Exp Cell Res 315: 1653-1667.
29. Nishida T, Yamada Y (2008) SMT3IP1, a nucleolar SUMO-specific protease, deconjugates SUMO-2 from nucleolar and cytoplasmic nucleophosmin. Biochem Biophys Res Commun 374: 382-387.
30. Utama B, Kennedy D, Ru K, Mattick JS (2002) Isolation and characterization of a new nucleolar protein, Nrap, that is conserved from yeast to humans. Genes Cells 7: 115-132.
31. Hirano Y, Ishii K, Kumeta M, Furukawa K, Takeyasu K, et al. (2009) Proteomic and targeted analytical identification of BXDC1 and EBNA1BP2 as dynamic scaffold proteins in the nucleolus. Genes Cells 14: 155-166.
32. Holmstrom TH, Mialon A, Kallio M, Nymalm Y, Mannermaa L, et al. (2008) c-Jun supports ribosomal RNA processing and nucleolar localization of RNA helicase DDX21. J Biol Chem 283: 7046-7053.
33. Yang H, Henning D, Valdez BC (2005) Functional interaction between RNA helicase II/Gu(alpha) and ribosomal protein L4. FEBS J 272: 3788-3802.
34. Granneman S, Gallagher JE, Vogelzangs J, Horstman W, van Venrooij WJ, et al. (2003) The human Imp3 and Imp4 proteins form a ternary complex with hMpp10, which only interacts with the U3 snoRNA in 60-80S ribonucleoprotein complexes. Nucleic Acids Res 31: 1877-1887.
35. Damianov A, Kann M, Lane WS, Bindereif A (2006) Human RBM28 protein is a specific nucleolar component of the spliceosomal snRNPs. Biol Chem 387: 1455-1460.
36. Henras A, Henry Y, Bousquet-Antonelli C, Noaillac-Depeyre J, Gelugne JP, et al. (1998) Nhp2p and Nop10p are essential for the function of H/ACA snoRNPs. EMBO J 17: 7078-7090.
37. Su L, Hershberger RJ, Weissman IL (1993) LYAR, a novel nucleolar protein with zinc finger DNA-binding motifs, is involved in cell growth regulation. Genes Dev 7: 735-748.
38. Zirwes RF, Eilbracht J, Kneissel S, Schmidt-Zachmann MS (2000) A novel helicase-type protein in the nucleolus: protein NOH61. Mol Biol Cell 11: 1153-1167.
39. Michalec B, Krokowski D, Grela P, Wawiorka L, Sawa-Makarska J, et al. (2010) Subcellular localization of ribosomal P0-like protein MRT4 is determined by its N-terminal domain. Int J Biochem Cell Biol 42: 736-748.
40. Hasina R, Pontier AL, Fekete MJ, Martin LE, Qi XM, et al. (2006) NOL7 is a nucleolar candidate tumor suppressor gene in cervical cancer that modulates the angiogenic phenotype. Oncogene 25: 588-598.
41. Gunawardena SR, Ruis BL, Meyer JA, Kapoor M, Conklin KF (2008) NOM1 targets protein phosphatase I to the nucleolus. J Biol Chem 283: 398-404.
42. Simmons HM, Ruis BL, Kapoor M, Hudacek AW, Conklin KF (2005) Identification of NOM1, a nucleolar, eIF4A binding protein encoded within the chromosome 7q36

breakpoint region targeted in cases of pediatric acute myeloid leukemia. Gene 347: 137-145.
43. Eilbracht J, Kneissel S, Hofmann A, Schmidt-Zachmann MS (2005) Protein NO52--a constitutive nucleolar component sharing high sequence homologies to protein NO66. Eur J Cell Biol 84: 279-294.
44. Tsai YT, Lin CI, Chen HK, Lee KM, Hsu CY, et al. (2008) Chromatin tethering effects of hNopp140 are involved in the spatial organization of nucleolus and the rRNA gene transcription. J Biomed Sci 15: 471-486.
45. Eilbracht J, Reichenzeller M, Hergt M, Schnolzer M, Heid H, et al. (2004) NO66, a highly conserved dual location protein in the nucleolus and in a special type of synchronously replicating chromatin. Mol Biol Cell 15: 1816-1832.
46. Rajendran RR, Nye AC, Frasor J, Balsara RD, Martini PG, et al. (2003) Regulation of nuclear receptor transcriptional activity by a novel DEAD box RNA helicase (DP97). J Biol Chem 278: 4628-4638.
47. Messaoudi L, Yang YG, Kinomura A, Stavreva DA, Yan G, et al. (2007) Subcellular distribution of human RDM1 protein isoforms and their nucleolar accumulation in response to heat shock and proteotoxic stress. Nucleic Acids Res 35: 6571-6587.
48. Du X, Rao MR, Chen XQ, Wu W, Mahalingam S, et al. (2006) The homologous putative GTPases Grn1p from fission yeast and the human GNL3L are required for growth and play a role in processing of nucleolar pre-rRNA. Mol Biol Cell 17: 460-474.
49. Lechertier T, Grob A, Hernandez-Verdun D, Roussel P (2009) Fibrillarin and Nop56 interact before being co-assembled in box C/D snoRNPs. Exp Cell Res 315: 928-942.
50. Ouyang P (2009) SRrp37, a novel splicing regulator located in the nuclear speckles and nucleoli, interacts with SC35 and modulates alternative pre-mRNA splicing in vivo. J Cell Biochem 108: 304-314.
51. Kruhlak M, Crouch EE, Orlov M, Montano C, Gorski SA, et al. (2007) The ATM repair pathway inhibits RNA polymerase I transcription in response to chromosome breaks. Nature 447: 730-734.
52. Zhu XD, Kuster B, Mann M, Petrini JH, de Lange T (2000) Cell-cycle-regulated association of RAD50/MRE11/NBS1 with TRF2 and human telomeres. Nat Genet 25: 347-352.
53. Kobayashi J, Tauchi H, Sakamoto S, Nakamura A, Morishima K, et al. (2002) NBS1 localizes to gamma-H2AX foci through interaction with the FHA/BRCT domain. Curr Biol 12: 1846-1851.
54. Woo LL, Futami K, Shimamoto A, Furuichi Y, Frank KM (2006) The Rothmund-Thomson gene product RECQL4 localizes to the nucleolus in response to oxidative stress. Exp Cell Res 312: 3443-3457.
55. Takemura M, Ohoka F, Perpelescu M, Ogawa M, Matsushita H, et al. (2002) Phosphorylation-dependent migration of retinoblastoma protein into the nucleolus triggered by binding to nucleophosmin/B23. Exp Cell Res 276: 233-241.
56. Yaneva M, Jhiang S (1991) Expression of the Ku protein during cell proliferation. Biochim Biophys Acta 1090: 181-187.
57. Komamura-Kohno Y, Karasawa-Shimizu K, Saitoh T, Sato M, Hanaoka F, et al. (2006) Site-specific phosphorylation of MCM4 during the cell cycle in mammalian cells. FEBS J 273: 1224-1239.
58. Szekely AM, Chen YH, Zhang C, Oshima J, Weissman SM (2000) Werner protein recruits DNA polymerase delta to the nucleolus. Proc Natl Acad Sci U S A 97: 11365-11370.
59. Yoo JE, Oh BK, Park YN (2009) Human PinX1 mediates TRF1 accumulation in nucleolus and enhances TRF1 binding to telomeres. J Mol Biol 388: 928-940.
60. Grummt I (2007) Different epigenetic layers engage in complex crosstalk to define the epigenetic state of mammalian rRNA genes. Hum Mol Genet 16 Spec No 1: R21-27.
61. Schilders G, van Dijk E, Pruijn GJ (2007) C1D and hMtr4p associate with the human exosome subunit PM/Scl-100 and are involved in pre-rRNA processing. Nucleic Acids Res 35: 2564-2572.
62. von Kobbe C, Bohr VA (2002) A nucleolar targeting sequence in the Werner syndrome protein resides within residues 949-1092. J Cell Sci 115: 3901-3907.
63. Dynes JL, Xu S, Bothner S, Lahti JM, Hori RT (2004) The carboxyl-terminus directs TAF(I)48 to the nucleus and nucleolus and associates with multiple nuclear import receptors. J Biochem 135: 429-438.

64. Santoro R, Li J, Grummt I (2002) The nucleolar remodeling complex NoRC mediates heterochromatin formation and silencing of ribosomal gene transcription. Nat Genet 32: 393-396.
65. Becherel OJ, Gueven N, Birrell GW, Schreiber V, Suraweera A, et al. (2006) Nucleolar localization of aprataxin is dependent on interaction with nucleolin and on active ribosomal DNA transcription. Hum Mol Genet 15: 2239-2249.
66. Vallee D, Chevrier E, Graham GE, Lazzaro MA, Lavigne PA, et al. (2004) A novel PHF6 mutation results in enhanced exon skipping and mild Borjeson-Forssman-Lehmann syndrome. J Med Genet 41: 778-783.
67. Olson MO, Dundr M, Szebeni A (2000) The nucleolus: an old factory with unexpected capabilities. Trends Cell Biol 10: 189-196.
68. Stoldt S, Wenzel D, Schulze E, Doenecke D, Happel N (2007) G1 phase-dependent nucleolar accumulation of human histone H1x. Biol Cell 99: 541-552.
69. Yu B, Mitchell GA, Richter A (2005) Nucleolar localization of cirhin, the protein mutated in North American Indian childhood cirrhosis. Exp Cell Res 311: 218-228.
70. Yu B, Mitchell GA, Richter A (2009) Cirhin up-regulates a canonical NF-kappaB element through strong interaction with Cirip/HIVEP1. Exp Cell Res 315: 3086-3098.
71. Lin DY, Shih HM (2002) Essential role of the 58-kDa microspherule protein in the modulation of Daxx-dependent transcriptional repression as revealed by nucleolar sequestration. J Biol Chem 277: 25446-25456.
72. Ren Y, Busch RK, Perlaky L, Busch H (1998) The 58-kDa microspherule protein (MSP58), a nucleolar protein, interacts with nucleolar protein p120. Eur J Biochem 253: 734-742.
73. Welsh GI, Kadereit S, Coccia EM, Hovanessian AG, Meurs EF (1999) Colocalization within the nucleolus of two highly related IFN-induced human nuclear phosphoproteins with nucleolin. Exp Cell Res 250: 62-74.
74. Wu H, Xu H, Miraglia LJ, Crooke ST (2000) Human RNase III is a 160-kDa protein involved in preribosomal RNA processing. J Biol Chem 275: 36957-36965.
75. Scott M, Boisvert FM, Vieyra D, Johnston RN, Bazett-Jones DP, et al. (2001) UV induces nucleolar translocation of ING1 through two distinct nucleolar targeting sequences. Nucleic Acids Res 29: 2052-2058.
76. Austin KM, Leary RJ, Shimamura A (2005) The Shwachman-Diamond SBDS protein localizes to the nucleolus. Blood 106: 1253-1258.
77. Zullo AJ, Michaud M, Zhang W, Grusby MJ (2009) Identification of the small protein rich in arginine and glycine (SRAG): a newly identified nucleolar protein that can regulate cell proliferation. J Biol Chem 284: 12504-12511.
78. Kawase T, Ichikawa H, Ohta T, Nozaki N, Tashiro F, et al. (2008) p53 target gene AEN is a nuclear exonuclease required for p53-dependent apoptosis. Oncogene 27: 3797-3810.
79. Shin C, Kleiman FE, Manley JL (2005) Multiple properties of the splicing repressor SRp38 distinguish it from typical SR proteins. Mol Cell Biol 25: 8334-8343.
80. Weber JD, Taylor LJ, Roussel MF, Sherr CJ, Bar-Sagi D (1999) Nucleolar Arf sequesters Mdm2 and activates p53. Nat Cell Biol 1: 20-26.
81. Dundr M, Hoffmann-Rohrer U, Hu Q, Grummt I, Rothblum LI, et al. (2002) A kinetic framework for a mammalian RNA polymerase in vivo. Science 298: 1623-1626.
82. Dhanda RS, Lindberg SR, Olsson I (2008) The human SIN3B corepressor forms a nucleolar complex with leukemia-associated ETO homologues. BMC Mol Biol 9: 8.
83. Maass N, Rosel F, Schem C, Hitomi J, Jonat W, et al. (2002) Amplification of the BCAS2 gene at chromosome 1p13.3-21 in human primary breast cancer. Cancer Lett 185: 219-223.
84. Scherl A, Coute Y, Deon C, Calle A, Kindbeiter K, et al. (2002) Functional proteomic analysis of human nucleolus. Mol Biol Cell 13: 4100-4109.
85. Galcheva-Gargova Z, Gangwani L, Konstantinov KN, Mikrut M, Theroux SJ, et al. (1998) The cytoplasmic zinc finger protein ZPR1 accumulates in the nucleolus of proliferating cells. Mol Biol Cell 9: 2963-2971.
86. Alcivar A, Hu S, Tang J, Yang X (2003) DEDD and DEDD2 associate with caspase-8/10 and signal cell death. Oncogene 22: 291-297.
87. Stegh AH, Schickling O, Ehret A, Scaffidi C, Peterhansel C, et al. (1998) DEDD, a novel death effector domain-containing protein, targeted to the nucleolus. EMBO J 17: 5974-5986.

88. Politz JC, Yarovoi S, Kilroy SM, Gowda K, Zwieb C, et al. (2000) Signal recognition particle components in the nucleolus. Proc Natl Acad Sci U S A 97: 55-60.
89. Korner CG, Wormington M, Muckenthaler M, Schneider S, Dehlin E, et al. (1998) The deadenylating nuclease (DAN) is involved in poly(A) tail removal during the meiotic maturation of Xenopus oocytes. EMBO J 17: 5427-5437.
90. Towbin H, Ramjoue HP, Kuster H, Liverani D, Gordon J (1982) Monoclonal antibodies against eucaryotic ribosomes. Use to characterize a ribosomal protein not previously identified and antigenically related to the acidic phosphoproteins P1/P2. J Biol Chem 257: 12709-12715.
91. Kiefer P, Dickson C (1995) Nucleolar association of fibroblast growth factor 3 via specific sequence motifs has inhibitory effects on cell growth. Mol Cell Biol 15: 4364-4374.
92. Henderson JE, Amizuka N, Warshawsky H, Biasotto D, Lanske BM, et al. (1995) Nucleolar localization of parathyroid hormone-related peptide enhances survival of chondrocytes under conditions that promote apoptotic cell death. Mol Cell Biol 15: 4064-4075.
93. Andersen JS, Lam YW, Leung AK, Ong SE, Lyon CE, et al. (2005) Nucleolar proteome dynamics. Nature 433: 77-83.
94. Andersen JS, Lyon CE, Fox AH, Leung AK, Lam YW, et al. (2002) Directed proteomic analysis of the human nucleolus. Curr Biol 12: 1-11.
95. Sun H, Tu X, Prisco M, Wu A, Casiburi I, et al. (2003) Insulin-like growth factor I receptor signaling and nuclear translocation of insulin receptor substrates 1 and 2. Mol Endocrinol 17: 472-486.
96. Tu X, Batta P, Innocent N, Prisco M, Casaburi I, et al. (2002) Nuclear translocation of insulin receptor substrate-1 by oncogenes and Igf-I. Effect on ribosomal RNA synthesis. J Biol Chem 277: 44357-44365.
97. Chan CC, Dostie J, Diem MD, Feng W, Mann M, et al. (2004) eIF4A3 is a novel component of the exon junction complex. Rna 10: 200-209.
98. Holzmann K, Gerner C, Poltl A, Schafer R, Obrist P, et al. (2000) A human common nuclear matrix protein homologous to eukaryotic translation initiation factor 4A. Biochem Biophys Res Commun 267: 339-344.
99. Koroleva OA, Calder G, Pendle AF, Kim SH, Lewandowska D, et al. (2009) Dynamic behavior of Arabidopsis eIF4A-III, putative core protein of exon junction complex: fast relocation to nucleolus and splicing speckles under hypoxia. Plant Cell 21: 1592-1606.
100. Kakuk A, Friedlander E, Vereb G, Jr., Lisboa D, Bagossi P, et al. (2008) Nuclear and nucleolar localization signals and their targeting function in phosphatidylinositol 4-kinase PI4K230. Exp Cell Res 314: 2376-2388.
101. Jakel S, Gorlich D (1998) Importin beta, transportin, RanBP5 and RanBP7 mediate nuclear import of ribosomal proteins in mammalian cells. EMBO J 17: 4491-4502.
102. Jin A, Itahana K, O'Keefe K, Zhang Y (2004) Inhibition of HDM2 and activation of p53 by ribosomal protein L23. Mol Cell Biol 24: 7669-7680.
103. Benezra M, Greenberg RS, Masur SK (2007) Localization of ZO-1 in the nucleolus of corneal fibroblasts. Invest Ophthalmol Vis Sci 48: 2043-2049.
104. Owens-Grillo JK, Czar MJ, Hutchison KA, Hoffmann K, Perdew GH, et al. (1996) A model of protein targeting mediated by immunophilins and other proteins that bind to hsp90 via tetratricopeptide repeat domains. J Biol Chem 271: 13468-13475.
105. Parrott AM, Walsh MR, Reichman TW, Mathews MB (2005) RNA binding and phosphorylation determine the intracellular distribution of nuclear factors 90 and 110. J Mol Biol 348: 281-293.
106. Ali M, Ramprasad VL, Soumittra N, Mohamed MD, Jafri H, et al. (2008) A missense mutation in the nuclear localization signal sequence of CERKL (p.R106S) causes autosomal recessive retinal degeneration. Mol Vis 14: 1960-1964.
107. Inagaki Y, Mitsutake S, Igarashi Y (2006) Identification of a nuclear localization signal in the retinitis pigmentosa-mutated RP26 protein, ceramide kinase-like protein. Biochem Biophys Res Commun 343: 982-987.
108. Tuson M, Garanto A, Gonzalez-Duarte R, Marfany G (2009) Overexpression of CERKL, a gene responsible for retinitis pigmentosa in humans, protects cells from apoptosis induced by oxidative stress. Mol Vis 15: 168-180.
109. Hao Z, Li X, Qiao T, Du R, Zhang G, et al. (2006) Subcellular localization of CIAPIN1. J Histochem Cytochem 54: 1437-1444.

110. Sutherland HG, Lam YW, Briers S, Lamond AI, Bickmore WA (2004) 3D3/lyric: a novel transmembrane protein of the endoplasmic reticulum and nuclear envelope, which is also present in the nucleolus. Exp Cell Res 294: 94-105.
111. Milewska M, McRedmond J, Byrne PC (2009) Identification of novel spartin-interactors shows spartin is a multifunctional protein. J Neurochem 111: 1022-1030.
112. Lindsay AJ, McCaffrey MW (2009) Myosin Vb localises to nucleoli and associates with the RNA polymerase I transcription complex. Cell Motil Cytoskeleton 66: 1057-1072.
113. Pintucci G, Yu PJ, Saponara F, Kadian-Dodov DL, Galloway AC, et al. (2005) PDGF-BB induces vascular smooth muscle cell expression of high molecular weight FGF-2, which accumulates in the nucleus. J Cell Biochem 95: 1292-1300.
114. Sheng Z, Lewis JA, Chirico WJ (2004) Nuclear and nucleolar localization of 18-kDa fibroblast growth factor-2 is controlled by C-terminal signals. J Biol Chem 279: 40153-40160.
115. Sheng Z, Liang Y, Lin CY, Comai L, Chirico WJ (2005) Direct regulation of rRNA transcription by fibroblast growth factor 2. Mol Cell Biol 25: 9419-9426.
116. Taverna S, Rigogliuso S, Salamone M, Vittorelli ML (2008) Intracellular trafficking of endogenous fibroblast growth factor-2. FEBS J 275: 1579-1592.
117. Brooks WS, Banerjee S, Crawford DF (2007) G2E3 is a nucleo-cytoplasmic shuttling protein with DNA damage responsive localization. Exp Cell Res 313: 665-676.
