# Supplementary material for: PNAC: a protein nucleolar association classifier
Source: BMC Genomics. 2011 Jan 27;12:74. doi: 10.1186/1471-2164-12-74 (PMC3038921; doi:10.1186/1471-2164-12-74)
Supplement: Additional file 3 — SILAC-derived dataset. This list displays the SILAC-derived dataset protein identifiers and abundance ratios. [file 1471-2164-12-74-S3.PDF]

SILAC-derived dataset used to test PNAC

IPI accession	Nucleolar vs cytoplasmic ratio	Nucleoplasmic vs cytoplasmic ratio
IPI00010845	1.70E+00	4.60E+00
IPI00220739	6.10E-01	2.80E+00
IPI00012149	5.40E+00	8.00E-01
IPI00012575	2.80E-01	1.20E-01
IPI00022430	5.00E-03	6.50E-02
IPI00023004	1.70E-01	2.00E-01
IPI00024719	3.00E-01	1.80E-01
IPI00298961	1.10E-01	4.40E-01
IPI00005161	4.40E-01	3.80E-01
IPI00005162	4.50E-01	3.10E-01
IPI00011200	9.00E-02	1.20E-01
IPI00012074	8.10E-01	4.20E+00
IPI00305978	8.30E-02	2.40E-01
IPI00013468	4.80E-01	1.80E+00
IPI00013808	1.60E-01	7.20E-01
IPI00646917	2.20E+00	4.20E+00
IPI00029737	4.80E-01	2.00E+00
IPI00514622	3.00E-01	4.90E-01
IPI00219726	3.60E-01	2.80E+00
IPI00027569	1.40E+00	1.50E+01
IPI00299254	2.50E-01	2.10E-01
IPI00032406	9.20E-02	3.10E-01
IPI00024403	5.30E-02	1.50E-01
IPI00031564	1.50E-01	9.00E-02
IPI00289334	1.60E-01	5.90E-01
IPI00025366	1.20E+00	3.10E+00
IPI00337386	1.10E+00	3.30E+00
IPI00411426	8.20E-02	1.70E-01
IPI00025796	1.30E+00	4.00E+00
IPI00026087	8.00E-01	7.10E+00
IPI00026496	9.40E+00	9.70E-01
IPI00420014	6.40E-01	3.70E+00
IPI00026625	6.10E-01	4.00E+00
IPI00027180	6.70E-02	3.30E-01
IPI00027223	1.10E-01	1.40E-01
IPI00015602	9.40E-01	4.00E+00
IPI00215966	5.40E+00	6.40E-01
IPI00007927	9.10E-01	5.10E-01
IPI00099463	4.10E+00	3.80E+00
IPI00294755	1.60E-01	1.80E+00
IPI00221338	1.10E-01	2.80E-01
IPI00219871	6.80E-02	1.20E+00
IPI00014053	1.40E+00	4.60E+00
IPI00607708	4.30E-02	7.40E-02
IPI00328415	5.90E-01	4.60E+00
IPI00016862	1.50E-01	2.10E-01
IPI00017672	7.20E-02	9.20E-02
IPI00218493	1.70E-01	6.80E-01
IPI00018342	3.10E-01	1.60E-01
IPI00384444	2.20E-01	4.60E+00
IPI00021405	2.70E+00	1.50E+01

IPI00216952	2.70E+00	1.50E+01
IPI00025512	7.40E-02	8.80E-02
IPI00025874	3.50E-01	3.10E+00
IPI00006482	3.50E-01	1.70E+00
IPI00414005	3.70E-01	1.50E+00
IPI00007188	1.50E+00	5.10E+00
IPI00219678	1.60E-01	2.40E-01
IPI00008529	4.20E-02	2.40E-01
IPI00303476	1.40E+00	4.40E+00
IPI00027463	1.20E-02	2.80E-02
IPI00010796	8.20E-02	9.60E-01
IPI00011654	1.10E-01	1.10E-01
IPI00216691	1.50E-02	2.60E-02
IPI00296053	6.20E-01	1.70E+00
IPI00027493	1.90E-01	1.60E+00
IPI00554777	3.00E-01	2.50E-01
IPI00029264	1.20E+00	3.50E+00
IPI00029267	8.50E-01	6.00E+00
IPI00290204	9.80E-01	6.80E+00
IPI00220578	1.20E-01	1.10E+00
IPI00015911	1.40E+00	3.80E+00
IPI00449049	2.30E+00	3.60E+00
IPI00418262	9.70E-02	3.00E-01
IPI00218448	3.80E+00	1.90E+01
IPI00217467	5.20E+00	1.40E+01
IPI00021338	1.70E+00	5.60E+00
IPI00021805	9.90E-01	3.80E+00
IPI00003865	4.20E-02	2.10E-01
IPI00003923	3.60E-01	2.60E-01
IPI00021700	7.20E-02	2.40E-01
IPI00022891	1.60E+00	5.10E+00
IPI00291467	1.50E+00	5.20E+00
IPI00644712	3.60E-01	3.00E-01
IPI00006579	1.20E+00	3.20E+00
IPI00186290	7.70E-02	1.20E-01
IPI00009904	1.80E-01	1.00E+00
IPI00010810	1.00E+00	4.00E+00
IPI00027230	1.30E-01	9.60E-01
IPI00216951	1.50E-01	2.00E-01
IPI00554711	2.30E-01	2.50E+00
IPI00220416	1.10E+00	3.50E+00
IPI00013485	5.10E-02	1.50E-01
IPI00013933	8.90E-01	2.30E+00
IPI00217182	8.10E-01	1.80E+00
IPI00219037	5.90E+00	2.00E+01
IPI00217466	5.40E+00	1.40E+01
IPI00219029	7.50E-02	9.10E-02
IPI00290566	6.40E-02	1.80E-01
IPI00029731	5.00E-01	2.20E-01
IPI00215918	7.30E-02	1.60E-01
IPI00219841	8.40E-02	3.70E-01
IPI00290077	2.80E-01	6.70E+00
IPI00291093	2.80E+00	8.60E-01
IPI00255316	7.40E+00	2.10E+01
IPI00025086	1.10E+00	4.20E+00
IPI00217975	1.50E+00	9.20E+00

IPI00218371	8.10E-02	9.90E-02
IPI00216308	9.40E-01	4.70E+00
IPI00645078	9.80E-02	1.50E-01
IPI00419983	6.90E+00	1.90E+01
IPI00396378	1.00E+00	1.20E+01
IPI00305383	1.50E+00	4.90E+00
IPI00010740	1.30E+01	2.80E+00
IPI00013871	3.10E-01	2.00E-01
IPI00028116	3.80E-01	3.20E+00
IPI00029133	1.70E+00	5.20E+00
IPI00030363	9.40E-01	3.40E+00
IPI00440493	1.60E+00	5.20E+00
IPI00219622	1.00E-01	1.30E-01
IPI00031556	7.70E-01	5.10E+00
IPI00219097	2.20E-01	7.70E-01
IPI00000875	6.80E-02	8.10E-02
IPI00018146	6.30E-02	1.10E-01
IPI00000811	2.40E-02	1.50E-01
IPI00400812	8.50E-01	3.40E+00
IPI00024919	9.90E-01	3.70E+00
IPI00024920	1.80E+00	4.90E+00
IPI00219953	3.10E+00	1.90E-01
IPI00219446	3.20E-02	6.80E-02
IPI00103467	7.30E-01	3.80E+00
IPI00012535	1.90E-01	2.70E-01
IPI00013847	1.10E+00	3.40E+00
IPI00289499	1.00E-01	1.40E-01
IPI00013877	9.50E-01	8.20E+00
IPI00216495	8.40E-01	8.30E+00
IPI00216318	8.60E-02	9.20E-02
IPI00013890	1.20E-01	1.40E-01
IPI00027350	1.90E-02	3.30E-02
IPI00550882	1.20E+00	3.20E+00
IPI00031691	3.10E-01	2.10E-01
IPI00002519	5.50E-01	9.80E-01
IPI00017334	1.40E+00	4.90E+00
IPI00017381	1.30E+00	8.30E-01
IPI00017412	1.50E+00	8.50E-01
IPI00219153	3.10E-01	2.10E-01
IPI00220219	2.00E-01	9.40E-01
IPI00021435	1.40E-01	2.10E-01
IPI00003328	4.50E-01	1.00E+00
IPI00395769	1.70E+00	4.20E+00
IPI00093057	1.80E-01	6.50E-01
IPI00005158	1.30E+00	3.90E+00
IPI00005705	1.40E+00	7.70E-01
IPI00420108	1.20E+00	3.50E+00
IPI00293434	9.60E-01	5.60E-01
IPI00550363	3.20E-01	1.40E-01
IPI00556451	1.60E+00	3.40E+00
IPI00007765	9.30E-01	3.20E+00
IPI00215780	6.80E-02	1.90E-01
IPI00550021	7.80E-01	3.40E-01
IPI00025849	5.60E-02	1.30E-01
IPI00026215	2.00E-01	6.70E-01
IPI00027626	5.70E-02	2.10E-01

IPI00291005	3.90E-02	8.30E-02
IPI00291006	1.10E+00	3.60E+00
IPI00031522	1.20E+00	3.40E+00
IPI00300567	9.70E-01	3.10E+00
IPI00647217	5.80E+00	1.40E+00
IPI00513971	1.00E-01	2.30E-01
IPI00412607	4.10E-01	2.10E-01
IPI00017895	8.00E-01	2.70E+00
IPI00301021	2.50E-01	3.80E+00
IPI00020042	1.10E-01	1.80E-01
IPI00024145	1.10E+00	3.90E+00
IPI00024674	3.60E-01	1.20E-01
IPI00004233	6.70E+00	1.20E+00
IPI00413173	7.00E+00	1.20E+00
IPI00294891	1.60E+01	1.30E+00
IPI00182533	2.50E-01	2.60E-01
IPI00221088	1.90E-01	2.10E-01
IPI00009342	2.10E-01	2.80E-01
IPI00026964	1.80E+00	4.50E+00
IPI00007611	1.30E+00	4.40E+00
IPI00008215	7.10E-02	1.30E-01
IPI00010720	6.30E-02	1.80E-01
IPI00183400	6.90E-01	7.00E-01
IPI00011134	2.00E-02	2.40E-01
IPI00027107	1.60E+00	3.20E+00
IPI00221234	2.80E-01	4.30E-01
IPI00030702	1.40E+00	5.10E+00
IPI00032140	3.60E-01	1.60E+00
IPI00002255	1.80E-01	2.40E-01
IPI00003021	3.50E-01	1.60E+00
IPI00419373	9.90E-01	8.10E+00
IPI00219525	6.40E-02	1.00E-01
IPI00003309	7.10E-01	2.50E-01
IPI00005969	1.50E-01	2.00E-01
IPI00294159	1.30E+00	3.80E+00
IPI00295857	2.90E-01	7.90E-01
IPI00004795	1.20E-01	1.60E-01
IPI00008161	8.50E-01	1.70E+00
IPI00218989	1.50E-01	5.10E-01
IPI00008982	2.00E+00	4.80E+00
IPI00022648	5.60E-01	1.20E-01
IPI00023860	1.00E-01	1.00E-01
IPI00394665	7.00E+00	3.70E+00
IPI00008240	1.50E-01	1.90E-01
IPI00218848	8.90E-01	2.70E+00
IPI00010105	1.90E+00	2.60E-01
IPI00477495	6.60E+00	2.40E+01
IPI00299084	6.50E-02	3.00E+00
IPI00027970	8.60E-02	3.40E-01
IPI00152906	1.10E+01	3.00E+01
IPI00554811	2.90E-01	3.40E-01
IPI00451401	4.50E-03	3.10E-02
IPI00021439	8.10E-02	4.80E-01
IPI00028481	1.70E-01	1.40E+00
IPI00300299	4.50E-01	3.80E+00
IPI00003949	5.00E-02	7.80E-02

IPI00028091	3.30E-01	3.70E-01
IPI00215917	5.20E-02	1.30E-01
IPI00015148	7.60E-02	1.30E+00
IPI00027270	5.60E-01	3.00E-01
IPI00219155	6.40E-01	2.20E-01
IPI00414860	1.30E-01	2.00E-01
IPI00220362	9.80E-01	3.00E+00
IPI00216049	5.00E-01	2.60E+00
IPI00550451	9.80E-01	1.00E+00
IPI00218236	1.40E+00	7.10E-01
IPI00075248	1.20E-01	4.00E-01
IPI00023919	1.10E-01	3.40E-01
IPI00221092	2.30E-01	2.20E-01
IPI00000816	6.50E-02	1.80E-01
IPI00026271	5.00E-01	2.80E-01
IPI00218606	2.60E-01	2.30E-01
IPI00221089	3.70E-01	2.10E-01
IPI00029266	5.80E-01	3.40E+00
IPI00016572	8.90E-01	4.20E+00
IPI00017963	4.60E-01	2.60E+00
IPI00429191	2.00E-01	1.90E-01
IPI00217030	5.20E-01	3.00E-01
IPI00429689	9.20E-02	1.30E-01
IPI00012750	3.80E-02	1.90E-01
IPI00719622	1.80E-01	2.00E-01
IPI00219156	4.90E-01	2.80E-01
IPI00395998	5.60E-01	3.10E-01
IPI00019329	9.60E-02	9.80E-01
IPI00215790	2.40E-01	2.70E-01
IPI00023006	6.10E-02	4.30E-01
IPI00007750	2.90E-02	8.50E-02
IPI00026546	8.10E-02	5.30E-02
IPI00019196	6.00E+00	8.40E-01
IPI00296337	7.40E+00	2.90E+00
IPI00306332	2.10E-01	2.40E-01
IPI00215919	5.70E-02	1.70E-01
IPI00025329	4.30E-01	2.40E-01
IPI00024320	7.60E-01	1.60E+00
IPI00022202	1.20E+00	4.80E+00
IPI00216057	1.10E-01	7.40E-02
IPI00009790	1.40E-01	1.90E-01
IPI00026202	5.50E-01	3.20E-01
IPI00029744	1.30E+00	3.80E+00
IPI00014424	1.10E-01	1.80E-01
IPI00029534	1.20E-01	9.20E-02
IPI00215719	6.70E-01	3.00E-01
IPI00014230	1.10E+00	3.00E+00
IPI00019380	4.90E-01	2.20E+00
IPI00373870	9.70E-01	6.90E+00
IPI00005198	1.30E+00	2.10E+00
IPI00030275	7.90E-01	2.80E+00
IPI00412298	2.00E+00	9.70E-01
IPI00156032	2.00E+01	7.70E-01
IPI00179709	5.80E-02	9.00E-02
IPI00015905	5.60E+00	8.00E-01
IPI00028275	3.90E-02	2.00E-01

IPI00028980	7.50E+00	1.00E+00
IPI00029012	1.40E-01	2.80E-01
IPI00456969	1.60E-01	2.80E-01
IPI00032304	1.20E-01	6.70E-01
IPI00291939	2.10E+00	3.10E+00
IPI00006099	9.50E+00	8.80E-01
IPI00472939	5.60E-01	3.60E+00
IPI00014177	3.40E-01	5.30E-01
IPI00003519	5.40E-01	3.10E+00
IPI00014238	1.40E-01	1.80E-01
IPI00008599	3.00E-01	3.20E+00
IPI00329352	2.20E-01	2.30E+00
IPI00304596	9.20E+00	2.00E+00
IPI00300078	1.70E+01	1.50E+00
IPI00300371	6.00E-01	4.00E+00
IPI00019472	2.20E-01	1.20E+00
IPI00009946	2.20E-01	2.30E-01
IPI00020436	8.80E-02	6.80E-01
IPI00003377	2.20E+00	1.10E+01
IPI00003406	3.80E-01	1.90E+00
IPI00163187	6.90E-02	2.20E-01
IPI00003482	9.30E-01	2.80E+00
IPI00419844	6.10E-01	2.80E+00
IPI00101186	1.40E+01	1.20E+00
IPI00294794	1.50E+01	1.20E+00
IPI00306048	9.80E-01	2.60E+00
IPI00007928	7.40E-01	4.50E+00
IPI00140420	1.40E-01	7.90E-01
IPI00100160	2.20E-01	2.70E-01
IPI00216730	4.60E+00	1.50E+01
IPI00217240	9.40E+00	1.00E+00
IPI00217630	7.00E+00	1.60E+00
IPI00169325	1.30E+01	1.20E+00
IPI00219950	4.60E-02	1.80E-01
IPI00152692	1.50E-01	2.60E-01
IPI00152708	7.60E+00	7.20E-01
IPI00102815	1.00E+01	9.80E-01
IPI00103599	9.00E-01	2.30E+00
IPI00293655	2.20E-01	7.20E-01
IPI00514983	1.30E-01	9.40E-02
IPI00001159	2.60E-01	3.00E-01
IPI00020194	1.40E+00	3.10E+00
IPI00025347	1.10E+01	9.40E-01
IPI00056357	6.80E-02	8.40E-01
IPI00470610	1.30E+00	3.40E+00
IPI00647161	2.90E-02	1.10E+00
IPI00081836	5.40E+00	1.80E+01
IPI00185146	1.70E-01	1.10E-01
IPI00013174	7.70E+00	2.60E+00
IPI00045109	5.10E+00	1.80E+01
IPI00298547	2.10E-02	8.00E-02
IPI00293350	2.00E-02	9.30E-02
IPI00329629	3.00E-01	4.60E-01
IPI00106509	5.20E-01	6.30E+00
IPI00018465	1.10E-01	2.20E-01
IPI00027831	2.10E+00	3.90E-01

IPI00029513	7.50E+00	8.60E-01
IPI00165393	5.50E-02	6.10E-02
IPI00107113	1.00E+01	1.00E+00
IPI00306380	1.10E+01	9.10E-01
IPI00010404	5.50E-01	5.40E+00
IPI00385042	1.10E+01	9.80E-01
IPI00006987	1.10E+01	1.10E+00
IPI00100151	4.20E+00	8.10E-01
IPI00008964	9.00E-02	1.00E+00
IPI00024279	1.40E+01	1.20E+00
IPI00303813	1.10E+01	9.30E-01
IPI00418797	5.90E+00	6.30E-01
IPI00029557	1.10E+00	3.10E+00
IPI00444452	1.40E-01	2.70E-01
IPI00015955	9.40E+00	1.20E+00
IPI00300074	1.60E-01	1.60E-01
IPI00328298	5.70E-01	3.40E-01
IPI00216105	1.10E+00	1.10E-01
IPI00167941	3.60E+00	1.60E+00
IPI00020128	8.60E+00	9.90E-01
IPI00170692	4.90E-01	1.80E+00
IPI00465044	9.70E-01	2.10E+00
IPI00002557	1.00E-01	2.00E+00
IPI00170796	7.30E-02	2.50E-01
IPI00037448	1.70E-01	2.90E-01
IPI00554701	4.20E-01	1.30E+00
IPI00008248	7.30E-01	2.40E+00
IPI00009057	2.70E-01	1.60E-01
IPI00009235	1.10E-01	4.40E+00
IPI00009471	1.10E+01	1.10E+00
IPI00299000	1.50E-01	1.50E-01
IPI00219420	2.00E+00	3.00E+00
IPI00006980	2.00E-01	8.40E-01
IPI00009104	2.10E-01	4.80E-01
IPI00021187	1.30E-01	3.40E-01
IPI00031820	1.90E-01	2.50E-01
IPI00032823	3.40E+00	4.40E-01
IPI00032903	1.10E+00	2.90E+00
IPI00411886	9.70E+00	9.50E-01
IPI00298994	2.10E-01	2.70E-01
IPI00000733	1.00E+01	1.10E+00
IPI00001757	1.90E-01	2.40E+00
IPI00102069	4.90E-02	1.30E-01
IPI00382804	8.30E-02	1.30E-01
IPI00719047	2.70E-01	1.80E-01
IPI00719549	8.60E+00	3.20E+00
IPI00377005	6.40E-02	1.40E-01
IPI00604590	4.40E-02	4.60E-02
IPI00655812	2.90E+00	1.50E+01
IPI00411329	1.10E+00	9.80E+00
IPI00479946	6.90E-01	1.50E-01
IPI00604504	3.10E+01	8.80E-01
IPI00105598	9.70E-02	1.50E-01
IPI00556482	1.60E-01	1.30E+00
IPI00556538	5.30E-02	1.10E+00
IPI00555565	1.90E-02	4.20E-01

IPI00556013	9.50E-02	1.10E-01
IPI00556514	8.70E+00	3.10E+00
IPI00478327	1.90E-01	2.20E-01
IPI00470658	7.70E-01	4.70E+00
IPI00450975	2.20E-01	2.00E-01
IPI00025447	8.20E-02	1.20E-01
IPI00001661	1.40E+00	4.60E+00
IPI00418523	9.10E+00	8.70E-01
IPI00465179	1.20E-01	2.00E-01
IPI00446473	4.20E+00	1.60E+00
IPI00385082	2.10E+00	3.40E+00
IPI00376005	6.10E-02	9.00E-02
IPI00023647	1.40E-01	1.70E-01
IPI00328587	2.40E-02	8.90E-02
IPI00385786	5.70E-01	1.00E+01
IPI00382990	1.00E-01	2.80E-01
IPI00382958	2.40E-02	4.20E-02
IPI00386403	6.10E-01	3.80E-01
IPI00386435	1.40E+00	4.80E+00
IPI00062003	8.60E-01	2.80E+00
IPI00410034	1.20E-01	1.20E+00
IPI00076042	1.40E+00	4.30E+00
IPI00411639	4.30E-02	1.80E-01
IPI00412987	6.00E-02	1.80E-01
IPI00386679	1.50E+00	5.40E+00
IPI00031605	7.30E-01	3.80E+00
IPI00386354	2.90E-01	3.30E-01
IPI00020075	1.20E+00	3.70E+00
IPI00019447	6.40E-01	5.90E-01
IPI00014402	3.50E-02	9.50E-02
IPI00383751	7.70E-02	9.80E-01
IPI00384282	3.50E-02	1.40E-01
IPI00304082	2.30E-01	1.90E-01
IPI00180128	1.00E-01	1.90E-01
IPI00291755	1.20E+00	5.20E+00
IPI00218733	6.80E-02	2.40E-02
IPI00302925	1.70E-01	2.30E-01
IPI00328318	2.80E-01	4.80E-01
IPI00293464	1.80E-01	5.20E-01
IPI00180730	1.10E-01	1.80E-01
IPI00249267	3.70E+00	2.10E+01
IPI00396485	1.10E-01	1.80E-01
IPI00411624	1.10E+00	1.40E+01
IPI00411633	4.80E-02	8.50E-02
IPI00413986	5.70E-01	2.20E-01
IPI00455482	3.90E-01	2.30E-01
IPI00477971	1.50E+01	1.20E+00
IPI00176696	1.50E-02	1.70E-01
IPI00398915	2.60E-01	2.40E-01
IPI00479743	4.50E-02	5.30E-01
IPI00397713	2.90E-01	2.50E-01
IPI00479281	7.50E-02	1.40E-01
IPI00478733	5.60E+00	2.50E+01
IPI00455757	4.40E-01	2.20E-01
IPI00477155	1.20E+01	1.10E+00
IPI00398057	1.20E-01	2.00E-01

IPI00024466	3.90E-01	1.40E+00
IPI00241841	7.80E-02	3.00E+00
IPI00219291	1.40E+00	4.10E+00
IPI00027423	1.10E+00	1.10E+00
IPI00290770	6.00E-02	1.90E-01
IPI00550591	9.40E-02	1.80E-01
IPI00552072	1.70E-01	3.00E-01
IPI00607772	2.80E-01	3.70E-01
IPI00479145	4.20E-01	8.60E+00
IPI00002459	1.70E-01	1.30E+00
IPI00013881	3.90E-01	3.80E+00
IPI00006440	1.40E+00	5.80E+00
IPI00171542	5.00E-01	3.00E+00
IPI00141938	4.00E+00	2.10E+01
IPI00383539	9.90E-01	3.00E+00
IPI00165486	5.00E-02	1.30E-01
IPI00036267	5.90E-01	4.40E-01
IPI00062151	5.20E-01	2.60E-01
IPI00035167	5.50E-01	2.20E-01
IPI00075558	5.10E-01	1.60E-01
IPI00175212	4.40E-01	2.40E-01
IPI00373807	5.40E+00	1.70E+01
IPI00457291	2.30E-01	1.90E-01
IPI00176678	6.60E-02	1.40E-01
IPI00738824	1.80E-01	2.70E-01
IPI00739952	3.30E-01	2.50E-01
IPI00742191	4.60E-01	2.00E-01
IPI00738381	8.50E-02	7.90E-02
IPI00738822	1.70E+00	1.50E+01
IPI00735397	5.10E-01	4.00E+00
IPI00738685	3.00E-01	1.80E-01
IPI00740142	5.90E-01	3.40E+00
IPI00742127	1.20E+00	1.20E+01
IPI00005585	1.00E-01	1.10E-01
IPI00016339	2.10E-01	1.10E+00
IPI00218200	1.90E-01	2.50E+00
IPI00031420	1.30E-01	1.40E-01
IPI00011698	1.80E+00	8.60E+00
IPI00007940	3.80E-01	2.60E+00
IPI00024067	9.00E-02	3.70E-01
IPI00306959	2.60E-01	5.20E+00
IPI00017367	6.10E-02	1.70E-01
IPI00646486	1.80E+00	4.60E+00
IPI00376798	4.30E-01	2.10E-01
IPI00647674	4.10E-01	1.70E-01
IPI00290410	3.60E+00	5.50E-01
IPI00103525	9.20E+00	1.80E+00
IPI00009943	6.10E-02	1.10E-01
IPI00552290	4.20E-02	1.30E-01
IPI00643196	1.00E-01	2.00E-01
IPI00552617	9.80E-02	2.00E-01
IPI00015671	1.70E-02	6.60E-02
IPI00645194	1.80E-01	8.50E-01
IPI00641692	4.90E+00	7.10E-01
IPI00640929	3.70E-01	3.40E-01
IPI00334627	4.30E-02	3.90E-01

IPI00515036	4.70E-01	2.20E-01
IPI00477040	1.00E+00	2.80E+00
IPI00017726	1.70E+00	3.80E+00
IPI00553151	2.00E+00	3.20E+00
IPI00376844	2.60E-02	4.70E-02
IPI00176692	1.00E+00	7.70E+00
IPI00014310	2.00E-01	6.00E-01
IPI00101659	6.50E+00	1.00E+00
IPI00295772	1.20E+00	2.90E+00
IPI00013508	1.20E-01	6.20E-01
IPI00009922	1.20E+00	3.50E+00
IPI00383680	5.10E-01	3.40E+00
IPI00552146	4.90E-01	2.60E+00
IPI00646493	2.80E-01	8.20E-01
IPI00639812	1.00E+00	5.00E+00
IPI00552308	2.50E-01	1.60E-01
IPI00642042	6.00E-02	1.30E-01
IPI00027165	4.00E-03	2.10E-02
IPI00641665	1.30E+00	1.80E+00
IPI00644722	3.30E-01	1.80E+00
IPI00640264	6.10E-02	9.90E-02
IPI00514910	5.70E-01	3.40E-01
IPI00552214	5.50E-01	4.20E+00
IPI00297241	8.10E+00	1.10E+00
IPI00641706	7.10E-02	1.00E-01
IPI00646946	6.60E-02	2.80E-01
IPI00644968	1.30E+00	9.40E+00
IPI00644037	4.90E-01	2.80E+00
IPI00171903	4.60E+00	5.50E+00
IPI00647546	1.80E-01	2.10E-01
IPI00217920	2.20E-01	1.40E-01
IPI00010201	1.10E-01	2.10E-01
IPI00303954	3.40E-01	5.00E+00
IPI00073602	6.90E+00	1.00E+00
IPI00383410	1.10E+01	1.00E+00
IPI00643087	1.20E+01	1.10E+00
IPI00291946	5.30E-01	4.80E-01
IPI00028031	9.80E-01	3.20E+00
IPI00884105	1.00E-01	8.40E-01
IPI00883870	4.50E-01	5.00E-01
IPI00883857	4.90E-01	3.70E+00
IPI00880053	1.50E+00	5.90E+00
IPI00879936	2.10E-01	5.40E+00
IPI00879599	5.80E-01	4.10E+00
IPI00879501	1.20E+00	9.50E+00
IPI00879437	7.40E-02	1.00E+00
IPI00879364	3.30E-01	2.50E-01
IPI00879148	5.50E-03	7.30E-02
IPI00879051	5.60E-02	6.30E-01
IPI00878827	7.00E-01	3.20E-01
IPI00878826	1.40E+00	2.90E-01
IPI00878524	8.40E-01	3.80E-01
IPI00878506	5.30E-01	2.30E-01
IPI00878470	4.50E-01	4.10E-01
IPI00878431	4.40E-01	1.90E-01
IPI00878369	8.90E-01	6.60E-01

IPI00878284	5.80E-02	1.80E-01
IPI00878272	8.80E-02	2.30E-01
IPI00878105	2.60E-01	2.40E-01
IPI00877922	4.10E-01	3.20E-01
IPI00876922	1.20E-01	1.40E-01
IPI00874178	4.70E-01	2.40E-01
IPI00873474	5.80E-02	1.70E-01
IPI00873444	1.10E-02	1.10E-01
IPI00873427	6.70E-02	9.00E-02
IPI00872901	3.00E+01	1.00E+00
IPI00872260	8.20E-01	8.00E+00
IPI00872214	3.30E-02	1.50E-01
IPI00872093	5.90E-01	3.70E-01
IPI00872079	8.60E-01	1.40E+01
IPI00871956	5.70E-02	1.70E-01
IPI00871777	9.40E-02	4.00E-01
IPI00871388	7.20E-01	3.80E+00
IPI00868835	1.20E+00	1.30E+01
IPI00856049	2.80E-01	2.40E-01
IPI00854687	9.70E-01	5.70E-01
IPI00852792	1.30E-01	9.10E-01
IPI00847774	5.50E-01	2.60E-01
IPI00847663	2.10E-01	1.70E+00
IPI00847192	1.90E-01	2.20E-01
IPI00844578	1.80E+01	3.70E+00
IPI00844375	2.70E-01	1.20E-01
IPI00844215	4.00E-01	2.10E+00
IPI00827508	5.90E-01	2.70E-01
IPI00815667	1.50E-01	2.20E-01
IPI00807522	1.00E-01	4.10E-01
IPI00798353	4.50E-02	1.20E-01
IPI00798341	1.10E-01	9.60E-02
IPI00798111	3.50E-02	3.80E-01
IPI00797902	1.20E+00	9.30E+00
IPI00797206	1.10E-01	1.80E-01
IPI00797126	6.00E-02	6.10E-02
IPI00796979	1.40E+00	2.70E+00
IPI00796881	4.10E-02	4.50E-01
IPI00796848	4.00E-01	1.10E+01
IPI00796689	8.10E+00	1.50E+00
IPI00796459	1.20E-01	3.60E-01
IPI00796102	3.10E-01	3.30E-01
IPI00795925	3.90E-02	3.20E-01
IPI00795717	2.90E-01	1.90E-01
IPI00795516	4.80E-02	8.80E-02
IPI00795109	1.20E-02	1.40E-01
IPI00794807	2.80E-01	4.60E+00
IPI00794644	5.20E-01	7.50E+00
IPI00794067	9.50E-02	5.80E-01
IPI00793917	1.90E-01	5.10E+00
IPI00793702	7.00E-01	4.50E+00
IPI00793498	1.10E-01	1.30E-01
IPI00793443	2.20E-01	2.80E-01
IPI00793381	8.40E-02	1.20E-01
IPI00793375	1.00E-01	7.50E-02
IPI00793285	1.10E-01	7.20E-01

IPI00793199	8.80E-02	1.20E-01
IPI00793186	5.30E-01	7.50E+00
IPI00792599	4.80E-01	1.80E+00
IPI00792534	1.30E+00	4.40E+00
IPI00792043	5.40E-01	1.70E+00
IPI00791418	4.20E-02	1.40E+00
IPI00791315	1.50E+00	5.80E+00
IPI00790847	1.00E+00	4.20E+00
IPI00790739	8.90E-01	2.60E+00
IPI00790725	1.40E+01	1.10E+00
IPI00790597	2.50E-02	1.10E-01
IPI00790432	9.00E-01	5.10E-01
IPI00790307	1.30E-01	1.00E-01
IPI00789135	1.50E+00	5.30E+00
IPI00789127	1.40E+00	1.10E+01
IPI00788782	3.00E-01	1.70E+00
IPI00787323	2.10E-01	4.70E+00
IPI00787105	5.10E-02	1.60E-01
IPI00787097	6.90E-01	2.80E-01
IPI00784154	1.50E+00	5.00E+00
IPI00783378	1.60E-01	2.30E-01
IPI00783271	1.20E+00	3.60E+00
IPI00748954	1.40E+01	5.20E+01
IPI00748298	1.60E-02	5.40E-02
IPI00748256	7.70E-02	1.50E-01
IPI00748057	1.20E-01	4.20E+00
IPI00747078	1.00E-01	1.40E+00
IPI00746694	2.20E-01	2.70E-01
IPI00745613	5.00E+00	7.20E-01
IPI00744706	3.30E-01	2.00E+00
IPI00744451	6.40E-02	1.60E-01
IPI00743305	1.30E-01	1.90E-01
IPI00742780	1.10E-01	1.70E-01
