# Supplementary material for: PNAC: a protein nucleolar association classifier
Source: BMC Genomics. 2011 Jan 27;12:74. doi: 10.1186/1471-2164-12-74 (PMC3038921; doi:10.1186/1471-2164-12-74)
Supplement: Additional file 4 — Description of discretization of SILAC abundance ratios into PNAC categories. This file lists the SILAC proteins used to define the SILAC ratio thresholds and displays the thresholds. [file 1471-2164-12-74-S4.PDF]

Dataset used to determine the abundance ratio thresholds defining the nucleolar association classes for the SILAC-derived dataset

Accession	Nucleolar vs cytoplasmic ratio	Nucleolar vs nucleoplasmic ratio	Literature-curated nucleolar association class
NP_001036100	5.67	3.66	Nucleolar-enriched
NP_115570	15.1	12.9	Nucleolar-enriched
NP_078938	11.4	9.19	Nucleolar-enriched
NP_057267	5.85	7.45	Nucleolar-enriched
NP_778224	7.13	0.308	Nucleolar-nucleoplasmic
NP_006017	2.77	0.412	Nucleolar-nucleoplasmic
NP_036555	0.484	1.94	Nucleolar-cytoplasmic
NP_001019833	0.651	2.21	Nucleolar-cytoplasmic
NP_001030168	0.458	2.11	Nucleolar-cytoplasmic
NP_000967	0.411	1.86	Nucleolar-cytoplasmic
NP_000981	0.553	1.55	Nucleolar-cytoplasmic
NP_001000	0.470	1.80	Nucleolar-cytoplasmic
NP_072045	0.115	0.540	Nucleolar-cytoplasmic
NP_055555	0.83	0.919	Nucleolar-cytoplasmic

To map SILAC abundance ratios into PNAC nucleolar-association groups (to be able to test PNAC using this SILAC-derived dataset), the following thresholds were used:

-nucleolar-enriched group:
nucleolar vs cytoplasmic ratio > 4.5
AND nucleolar vs nucleoplasmic ratio > 3.5

-nucleolar-nucleoplasmic group:
nucleoplasmic vs cytoplasmic ratio > 3.0
AND nucleolar vs cytoplasmic ratio > 2.0
AND nucleolar vs nucleoplasmic ratio > 0.2
AND nucleolar vs nucleoplasmic ratio < 1.0

-nucleolar-cytoplasmic group:
nucleoplasmic vs cytoplasmic ratio < 1.0
AND nucleolar vs cytoplasmic ratio > 0.2
AND nucleolar vs cytoplasmic ratio < 0.85
AND nucleolar vs nucleoplasmic ratio > 0.85
AND nucleoplasmic vs cytoplasmic ratio > 0.2

-non-nucleolar group
(nucleolar vs cytoplasmic ratio < 1.75 AND nucleolar vs nucleoplasmic ratio < 0.6)
OR nucleolar vs cytoplasmic ratio < 0.08
OR nucleolar vs nucleoplasmic ratio < 0.1
