# Supplementary material for: PNAC: a protein nucleolar association classifier
Source: BMC Genomics. 2011 Jan 27;12:74. doi: 10.1186/1471-2164-12-74 (PMC3038921; doi:10.1186/1471-2164-12-74)

Distribution of targeting motif scores

As described in the Methods section, the targeting motif module classifies proteins based on the presence of protein targeting motifs (signal peptides, transmembrane domains, mitochondrial targeting peptides and nucleolar localization sequences). Three scores were defined to represent the presence or absence of these motifs in proteins:

Mitochondrial score $s_M = 1$ if the protein encodes a predicted mitochondrial targeting peptide, $s_M = 0$ otherwise.

Secretory-membrane score $s_S = 1$ if the protein encodes a predicted signal peptide or at least one TMD, $s_S = 0$ otherwise.

NoLS score $s_N = 2$ if the maximum NoLS score output by NoD (see Methods) is >= 0.9.
$s_N = 1$ if the maximum NoLS score output by NoD is between 0.8 and 0.9.
$s_N = 0$ if the maximum NoLS score output by NoD is < 0.8.

The cyto score s_C is defined based on s_M and s_S such that $s_C = 2$ if $s_S = 1$ regardless of s_M, $s_C = 1$ if $s_M = 1$ and $s_S = 0$ and $s_C = 0$ if $s_M = 0$ and $s_S = 0$.

These s_C and s_N scores were grouped into nine bins representing all possible combinations of their states. Their distribution is plotted below, as an average of 5 independent runs (error bars represent standard deviation):

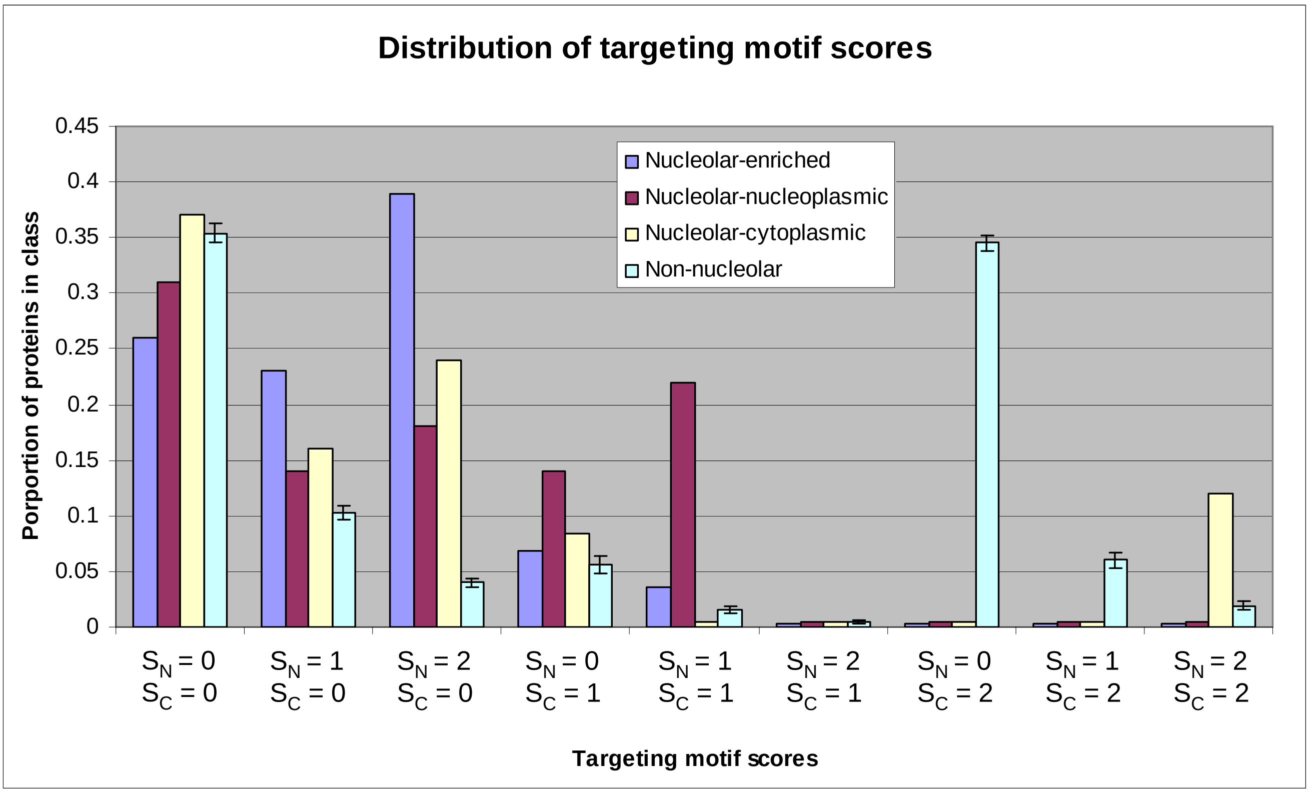

Supplement: Additional file 5 — Distribution of protein targeting motif scores per class. This file displays a plot of the distribution of scores for the targeting motif module. [file 1471-2164-12-74-S5.PDF]
